# Enhanced Recovery of Bioactive Compounds from *Rosa canina* L. Leaves: A Cascade Approach Using Ultrasounds and High-Pressure Homogenization

**DOI:** 10.3390/antiox15050560

**Published:** 2026-04-28

**Authors:** Zhanar Nabiyeva, Serena Carpentieri, Akerke Kulaipbekova, Abdyssemat Samadun, Yuliya Pronina, Elmira Assembayeva, Giovanna Ferrari

**Affiliations:** 1 Research Institute of Food Safety, Almaty Technological University, Almaty 050012, Kazakhstan; atu_nabiyeva@mail.ru (Z.N.); abdu.93_93@mail.ru (A.S.); 2 Department of Applied Biotechnology, Almaty Technological University, Almaty 050012, Kazakhstan; a.kulaipbekova@atu.edu.kz; 3 Department of Industrial Engineering, University of Salerno, 84084 Fisciano, Italy; gferrari@unisa.it; 4 Department of Food Technology, Almaty Technological University, Almaty 050012, Kazakhstan; medvezhonok_87@inbox.ru; 5 ProdAl Scarl, University of Salerno, 84084 Fisciano, Italy

**Keywords:** *Rosa canina* L. leaves, valorization, ultrasound-assisted extraction (UAE), high-pressure homogenization (HPH), cascade extraction, bioactive compounds

## Abstract

Background: This study proposes a cascade strategy for the comprehensive valorization of *Rosa canina* L. leaves, considered an underutilized agricultural by-product. Methods: The approach is based on a combination of optimized Ultrasound-assisted extraction (UAE) followed by High-pressure homogenization (HPH) of the residual biomass from both whole and ground leaves. UAE parameters (temperature, process duration, and ethanol concentration) were optimized to maximize the yield of total phenolic content (TPC), total flavonoid content (TFC), and antioxidant activity (DPPH, FRAP). Results: The optimal conditions (55.5 °C, 69.7 min, 40.8% ethanol) yielded extracts with a high TPC (289.55 mg GAE/g) and TFC (177.88 mg CE/g), reducing the processing time by 22% while increasing the TPC yield by 31% compared to the conventional solid–liquid extraction (SLE). It was found that primary extraction from whole leaves is more efficient than extraction from ground leaves, suggesting that the energy-intensive preliminary grinding step could be eliminated. The application of HPH to the residual biomass provided a significant secondary release of bioactive compounds, exceeding high-shear mixing (HSM) by up to 1.5 times for whole leaves. Kinetic analysis showed a higher release of bioactive compounds from whole leaves compared to ground leaves. Conclusions: The proposed UAE + HPH cascade process is a sustainable approach, ensuring rational use of resources and a significant increase in the total yield of antioxidants from *Rosa canina* L. leaves. Overall, the study may contribute to the circular economy by promoting valorization of agricultural by-products through an energy-efficient, sustainable cascade approach.

## 1. Introduction

Recently, the growing demand for natural antioxidants and phenolic compounds has led to increased interest in the valorization of plant by-products, especially those obtained from medicinal and wild plants, for further use in the food, pharmaceutical, and cosmetic industries. Within the framework of the circular bioeconomy concept, agricultural by-products are considered a promising source of biologically active compounds (BACs) that contribute to improving resource efficiency and reducing environmental impact [1].

Rosehip (*Rosa canina* L.) is a wild fruit shrub of the Rosaceae family, widely distributed in Europe, Asia, North America, and the Middle East [2]. In Kazakhstan, the Rosa genus is represented by more than 20 wild species, which are distinguished by their high biodiversity and significant nutritional and medicinal potential [3]. It is known that the chemical composition of *Rosa canina* L. varies significantly depending on the species, region of growth, climatic conditions, stage of ripeness, agricultural techniques, and storage conditions [4].

The fruits of *Rosa canina* L. are well-studied raw materials and are characterized by a high content of ascorbic acid, phenolic compounds, and polyunsaturated fatty acids, which determines their pronounced antioxidant activity and functional value [5,6,7,8,9]. In this regard, the fruits of *Rosa canina* L. are widely used in the food industry and pharmacology. In contrast, the leaves and stems of *Rosa canina* L. remain insufficiently studied, despite the rich biochemical composition of these vegetative parts of the plant, which could represent an alternative source of BACs.

Kubczak et al. (2020) [10] showed that extracts from the leaves and branches of *Rosa canina* L. are characterized by a high content of phenolic compounds and pronounced antioxidant activity. Analysis by high-performance liquid chromatography (HPLC) revealed significant concentrations of p-coumaric acid, myricetin, ellagic acid, cyanidin, procyanidins, and quercetin, with a relatively stable level of salicylic acid. In a study by Saad et al. (2022) [11], it was found that phenolic extracts from the leaves and stems of *Rosa canina* L. exhibit moderate antimicrobial and herbicidal activity due to the presence of phenolic acids, in particular caffeic and ferulic acids. The leaves had a higher content of flavonoids and total phenols, while the stems were richer in tannins and lignins, which exert structural and protective functions. Recent evidence has highlighted that *Rosa canina* L. leaves and other agro-industrial by-products are rich in antioxidant phytochemical compounds, offering significant potential for applications in the food, pharmaceutical, and cosmetic industries [9,12,13]. This potential underscores the need to develop efficient extraction methods to ensure a successful recovery of bioactive compounds and a sustainable valorization of *Rosa canina* L. leaves.

Solid–liquid extraction (SLE) is a traditional, widely used method based on the contact between plant material and solvent, which enables the diffusion of intracellular compounds. However, SLE is often characterized by long extraction times, significant energy consumption, and excessive solvent usage. In the context of a circular bioeconomy, the valorization of plant-based residues requires the implementation of intensified extraction technologies, moving toward a cascading approach [1]. This strategy is essential because high-value compounds are frequently embedded within complex cellular matrices. A cascading approach, based on a combination of traditional methods, such as SLE and ultrasound-assisted extraction (UAE), with additional physical processing of residual biomass, allows for the sequential recovery of different fractions, optimizing energy efficiency and ensuring that every component of the biomass is utilized to its maximum potential before final disposal [14,15,16,17]. In this context, UAE, through cavitation, can destroy the cellular structure, accelerate mass transport processes and, consequently, increase the yield of target compounds under milder conditions (short time, moderate temperature and solvent usage) [18,19,20].

High-pressure homogenization (HPH) is considered an effective mechanical disintegration method that disrupts cell structures and increases the availability of intracellular compounds, and is increasingly recognized as a key technology in integrated processing strategies. According to Pirozzi and Donsì (2023) [21], the inclusion of HPH in extraction processes of bioactive compounds from different agri-food by-products allowed for a 10–20% increase in the yield of total phenolic compounds compared to conventional SLE, while improving environmental safety and energy efficiency. Several studies have confirmed that the use of HPH contributes to an increase in the extractability of phenolic compounds and antioxidant activity of extracts obtained from agri-food by-products and effectively integrates into cascade schemes for valorizing plant by-products [14,15,16,17].

While research on *Rosa canina* L. fruits has successfully demonstrated the potential of combining extraction techniques like UAE and enzyme-assisted extraction (EAE) [22], such integrated approaches remain limited for leaves. Furthermore, despite the proven effectiveness of HPH treatment for various plant matrices, the application of this technology to *Rosa canina* L. leaves, particularly within a cascading framework, has not yet been explored. This represents a significant research gap, as the synergy between UAE and HPH holds substantial potential. While UAE efficiently facilitates the release of low-molecular-weight bioactive compounds through cavitation, HPH can effectively disrupt the cellular structure, unlocking high-molecular-weight compounds that remain trapped within the matrix. Integrating these technologies in a sequential approach could enhance extraction yields from *Rosa canina* L. leaves compared to conventional methods and produce high-value functional ingredients.

In this regard, this study aimed to comprehensively valorize *Rosa canina* L. leaves by optimizing the extraction processes for total phenolic content (TPC), total flavonoid content (TFC), and antioxidant activity (DPPH, FRAP). To achieve this goal, the SLE and UAE processing parameters were optimized to maximize the extractability of phenolic compounds and antioxidants from both whole and ground leaves. The release kinetics of BACs from residual biomass obtained after the first extraction step, using HPH, were also evaluated.

## 2. Materials and Methods

### 2.1. Vegetal Matrix

In August 2024, *Rosa canina* L. leaves were harvested from the Ile-Alatau National Park located in Almaty, Kazakhstan. The leaves underwent a shaded drying process at ambient temperature until a moisture level below 8% was achieved. Following stabilization, the samples were kept in a dark and moisture-free environment before being shipped to the ProdAl Scarl laboratories in Fisciano, Italy. For the experimental trials, the leaves were categorised into two groups: whole (intact) leaves and ground leaves. The latter were processed using a Retsch GM 200 mill (Retsch GmbH, Haan, Germany) to obtain a particle size range of 0.5–1.0 mm. This ground material was preserved in sealed glass jars at 20–25 °C in a ventilated space away from light.

### 2.2. Chemicals

The chemicals and solvents utilized in the present work met analytical-grade specifications and were acquired from Sigma Aldrich (Steinheim, Germany).

### 2.3. Extraction Procedures

**Figure 1:**
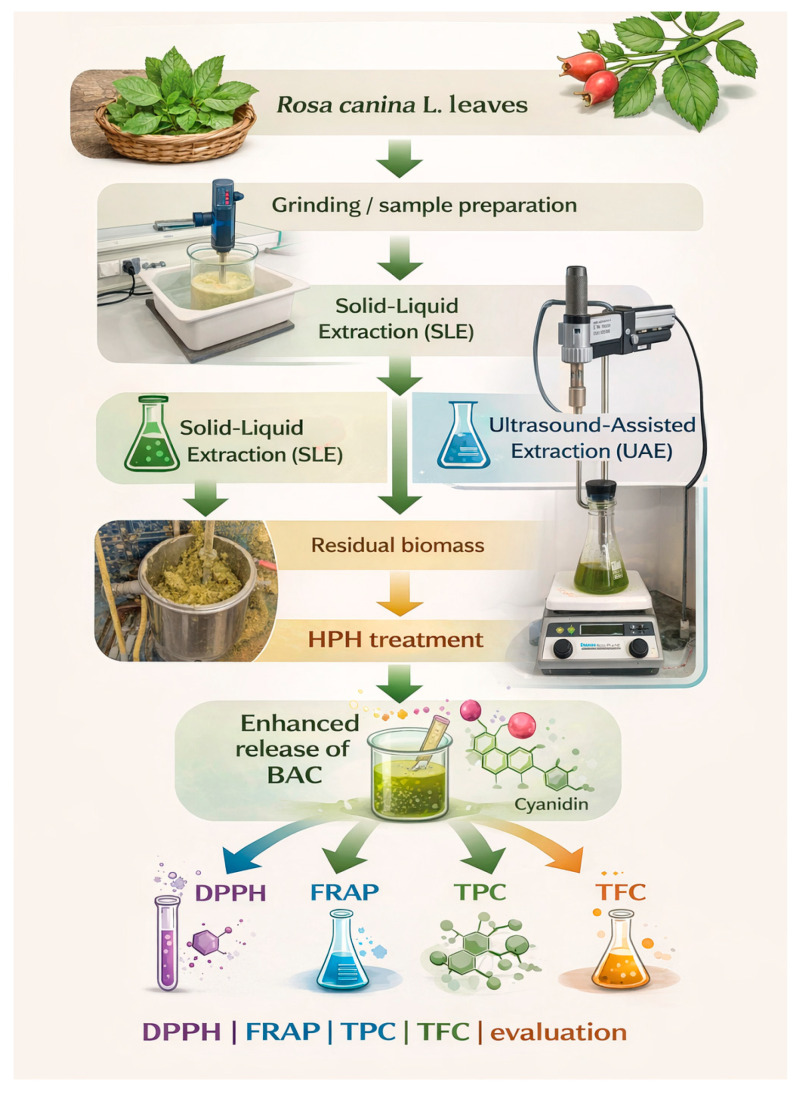
Experimental design and cascade extraction strategy used for the valorization of *Rosa canina* L. leaves, including solid–liquid extraction (SLE), ultrasound-assisted extraction (UAE), high-pressure homogenization (HPH), and evaluation of antioxidant activity (DPPH, FRAP), total phenolic content (TPC) and total flavonoid content (TFC). The figure was prepared using digital visualization tools, with AI-assisted support applied for graphical layout and clarity enhancement; the authors defined all scientific content.

The experimental design and cascade extraction strategy applied in this study are illustrated in Figure 1.

#### 2.3.1. First Extraction Stage of Bioactives from *Rosa canina* L. Leaves Using SLE and UAE

To determine the optimal operating conditions for SLE and UAE of both *Rosa canina* L. whole and ground leaves, different combinations of the extraction parameters were investigated to recover the highest concentration of bioactive compounds, specifically TPC, TFC, and antioxidant activity (FRAP and DPPH assays).

To optimize the extraction processes, Design Expert 12 software (Minneapolis, MN, USA) was used. A Face-Centred Central Composite Design (FC-CCD) was implemented to structure the experimental design. The experimental design included replicates of factorial and axial points to strengthen the model, with five replicates at the centre point.

The software enabled a comprehensive statistical evaluation of the experimental data by means of Analysis of Variance (ANOVA) to assess R^2^ values, adjusted R^2^, predicted R^2^, *p* values, lack of fits, and significance levels of individual model terms. Furthermore, the optimization of extraction parameters was achieved through desirability functions, followed by a validation of the predicted conditions. The optimal conditions obtained for each method and type of biomass (whole leaves and ground leaves) are given in Section 3.1 and were used in subsequent experiments.

##### Solid–Liquid Extraction (SLE)

In the case of the conventional SLE for rosehip whole leaves, a four-factor FC-CCD was used. Based on the outcomes of preliminary assays, the specific variables and their boundaries were established. The effects of the extraction time (10–90 min), temperature (25–70 °C), ethanol concentration in water (*v*/*v*) (0–80%), and solid-to-liquid (S/L) ratio (0.02–0.075 g/mL) on the response variables were analyzed. Based on preliminary experiments and literature data, in the case of the rosehip ground leaves, the S/L ratio was set at 0.05 g/mL. The obtained experimental designs are reported in Table 1 and Table 2 for rosehip whole and ground leaves, respectively.

The SLE process was performed by maceration using a magnetic stirring and heating plate (IKA^®^ C-MAG HS 7, IKA-Werke GmbH & Co. KG, Staufen im Breisgau, Germany) at a stirring rate of 300 rpm, as reported by Nabiyeva et al. (2025) [23].

##### Ultrasound-Assisted Extraction (UAE)

In the case of the UAE for rosehip whole and ground leaves, a three-factor FC-CCD was used. The S/L ratio was fixed at 0.072 g/mL and at 0.05 g/mL for rosehip whole and ground leaves, respectively, to reduce variability from this factor during the UAE process. The following parameter ranges were selected based on literature data and preliminary experiments: extraction time (30–90 min), temperature (40–70 °C), and ethanol concentration in water (*v*/*v*) (20–70%). The effects of these independent variables on TPC, TFC, FRAP, and DPPH values were investigated. The obtained experimental designs are reported in Table 3 and Table 4 for rosehip whole and ground leaves, respectively.

UAE was performed using a probe-type ultrasonic processor UP 400S (Hielscher GmbH, Chamerau, Germany), operating at a frequency of 24 kHz and a power of 100 W. The ultrasonic probe was immersed directly in the suspension, and the temperature was controlled using an external cooling bath.

For both SLE and UAE processes, a second-order polynomial model, reported in Equation (1), was used for fitting the obtained data.

(1) $$Y_{k}=\alpha_{0}+\sum_{i=1}^{4}\alpha_{i}X_{i}+\sum_{i=1}^{4}\alpha_{ii}X_{i}^{2}+\sum_{i=1}^{4}\sum_{j=i+1}^{4}\alpha_{ij}X_{i}X_{j}$$

where Y_k_ is the response; X_i_ and X_j_ are the input parameters; α_0_, α_i_, α_ii_, α_ij_ are the intercept and regression coefficients of the linear, quadratic, and interaction terms of the model, respectively.

#### 2.3.2. Second Extraction Stage of Bioactives from the Residual Biomass Using HPH

##### High-Pressure Homogenization (HPH)

The residual biomass of *Rosa canina* L. leaves following optimized SLE and UAE processes was suspended in deionized water at a concentration of 2% (*w*/*v*) and then homogenized using high-shear mixing (HSM) to ensure a uniform dispersion. HSM was performed as described by Carpentieri et al. (2023) [24] using a T–25 Ultra Turrax homogenizer (IKA^®^–Werke GmbH & Co. KG, Stuttgart, Germany) at 20,000 rpm for 10 min, while maintaining a constant temperature by immersing the device in an ice bath. This step was applied only at the initial time point (t = 0 min, 0 HPH passes) and provided a baseline for evaluating the effectiveness of the subsequent HPH treatment.

HPH was then applied using a laboratory-scale homogenizer [24]. Pressure regulation was managed through a system equipped with adaptable orifices (80–100 μm) from GmbH (Schweinfurt, Germany), which allowed for precise control over the pressure drop. The necessary pressure intensification was provided by a pneumatic Haskel pump, specifically the DXHF–683 model supplied by EGAR S.r.l. (Milan, Italy). Based on preliminary tests, experiments were conducted at a pressure of 80 MPa, 25 °C, and 0–10 recirculation passes (0–10 min). Thermal control was strictly maintained to prevent heating due to friction. This was achieved by cooling the jacketed feeding tank and utilizing two tube-in-tube heat exchangers before and after the valve. Samples were collected after 0, 5, and 10 min to evaluate the efficiency of cell wall disruption and residual bioactive compound extraction.

### 2.4. Analytical Methods

Quantitative determination of bioactive compounds and antioxidant activity was performed using a V-650 spectrometer (Jasco Inc., Easton, MD, USA), as previously described by Nabiyeva et al. (2025) [23].

#### 2.4.1. Total Phenolic Content (TPC)

The determination of TPC was carried out using the Folin–Ciocalteu method [23]. Absorbance was measured at 765 nm. TPC was quantified using gallic acid as a reference standard and expressed as mg gallic acid equivalents (GAE)/g dry weight (DW).

#### 2.4.2. Total Flavonoid Content (TFC)

TFC was determined using the aluminium chloride colourimetric assay. An aliquot of the samples was reacted with a 2% AlCl_3_ solution. Following an incubation period at room temperature, the absorbance was recorded at 430 nm. Results were reported as mg quercetin equivalents (QE)/g_DW_ [23].

#### 2.4.3. Antioxidant Activity

Antioxidant activity was assessed using two complementary assays: the ferric reducing antioxidant power (FRAP) and the DPPH (2,2-diphenyl-1-picrylhydrazyl) radical scavenging assay [23].

For the FRAP assay, absorbance readings were taken at 593 nm, and results were expressed as mg ascorbic acid equivalents (AAE)/g_DW_.

For the DPPH assay, the absorbance of the mixture was measured at 517 nm. The radical scavenging activity was calculated as a percentage of inhibition compared to the blank.

## 3. Results and Discussion

### 3.1. Model Validation and Extraction Efficiency

The statistical robustness of the experimental design was validated via ANOVA, confirming that the second-order polynomial models for TPC, TFC, FRAP, and DPPH were highly significant (*p* < 0.001) across all leaf morphologies and extraction techniques. The regression coefficients and statistical parameters are detailed in Tables S1–S4, while the Predicted vs. Experimental plots, depicted in Figures S3, S7, S11 and S15, illustrate a strong linear correlation. In all cases (Tables S1–S4), the Predicted R^2^ was in reasonable agreement with the Adjusted R^2^, with discrepancies consistently below the 0.2 threshold, demonstrating the high predictive power of the response surface methodology (RSM) employed. Furthermore, the non-significant Lack-of-Fit (*p* > 0.05) and low F-values across all responses statistically validate the adequacy of the quadratic models in describing the extraction dynamics of *Rosa canina* L. bioactive compounds. The normality of the data was graphically assessed through the Normal Probability Plots of Externally Studentized Residuals (Figures S4, S8, S12 and S16). For all investigated responses, the residuals follow a linear trend with no significant S-shaped patterns or extreme outliers. This distribution indicates that the normality assumption is satisfied and that the models provide a robust representation of the experimental data. The proximity of the points to the reference line further demonstrates the high accuracy of the regression equations in capturing the extraction dynamics.

These metrics indicate that the quadratic equations accurately predict the experimental data with minimal residual error, providing a superior framework for analyzing non-linear effects compared to traditional one-factor-at-a-time approaches [22,25]. Notably, the studied parameters significantly affected the bioactive indices, a finding consistent with previously published data on rosehip fruits and tea extracts [22,26]. The 3D response surfaces (Figures S1, S2, S5, S6, S9, S10, S13 and S14) elucidated distinct extraction dynamics according to the leaf morphology. For whole leaves, extraction was primarily diffusion-limited, with temperature (α_2_) and time (α_1_) acting as the main drivers for cellular wall softening and solute diffusion. Conversely, the linear term for ethanol concentration (α_3_) was often non-significant for TPC and TFC in whole leaves. As shown in Figures S1 and S5, maximum yields of TPC and TFC for whole leaves were reached at intermediate ethanol concentrations (32–40%) and moderate temperatures (~55 °C), following a trend in close agreement to that observed by Ilbay et al. (2013) [25], who identified ethanol concentration as a pivotal factor in the phenolic recovery from *Rosa canina* L. The negative quadratic coefficients for ethanol (α_33_) in these samples confirm a “bell-shaped” response; exceeding 40.8% ethanol likely shifts the solvent polarity away from the optimal range for compounds embedded within the intact leaf matrix. A significant shift in optimization profiles occurred in the case of ground leaves.

In this case, the linear term for ethanol (α_3_) became the dominant factor (*p* < 0.001), with optimal concentrations shifting toward higher ranges (78% for SLE and 68% for UAE). As illustrated in Figures S9 and S10, the response surfaces show a continuous upward trend as the ethanol concentration in water increases. This suggests that mechanical disruption eliminated structural barriers to mass transfer, shifting the rate-determining step from internal diffusion to thermodynamic solubility. The increased surface area of the powdered matrix facilitates the solubilization of less polar polyphenolic compounds. This transition is further corroborated by the significant positive interaction between time and ethanol (α13) (Table S3), implying that a concentrated ethanolic medium requires adequate contact time to fully penetrate and stabilize solutes within fine particles. The optimal S/L ratio also demonstrated a clear dependence on comminution. For whole leaves, the S/L ratio (α4) was not significant (Table S1), indicating that the solvent volume was sufficient to prevent saturation. Thus, a ratio of 0.072 g/mL was selected from Design Expert to minimize solvent consumption and maximize extract concentration, adhering to green chemistry principles. Conversely, for ground leaves, the S/L shifted to 0.05 g/mL [23]. This lower S/L ratio (higher relative solvent volume) is necessary to maintain a steep concentration gradient and prevent boundary layer saturation, which occurs rapidly when intracellular contents are immediately exposed to the solvent [27]. Finally, the impact of UAE was evident in the substantial reduction in processing time. For whole leaves, UAE achieved optimization in 69.7 min compared to 90 min for SLE, providing significantly higher TPC (289.55 vs. 220.70 mg GAE/g_DW_). In ground leaves, UAE reached its optimum in just 60 min at 68.2 °C. These findings are comparable to those found for other plant materials, such as *Psidium cattleianum* leaves (158.18 mg/g TPC) [28] and *Mucuna macrocarpa* leaves (186.61 mg GAE/g) [29], confirming the effectiveness of optimized ultrasound-assisted extraction to improve the recovery of bioactive compounds from leafy matrices, where temperature and solvent concentration played a decisive role in maximizing bioactivity [27].

This efficiency is attributed to acoustic cavitation, which induces cell wall disruption and micro-jets that accelerate mass transfer [25,26,27,28,29].

### 3.2. Optimal Conditions and Performance of the First-Step Extraction Methods

A central composite design was used to optimize the UAE and SLE processes for the recovery of BACs from *Rosa canina* L. leaves. Extraction efficiency was evaluated based on TPC, TFC, and antioxidant activity (FRAP and DPPH). For both SLE and UAE methods applied to whole and ground rosehip leaves, the optimal levels of the independent variables, identified through the desirability function to maximize all studied responses, the theoretical and empirical results for TPC, TFC, FRAP, and DPPH are summarized in Table 5, for whole leaves, and Table 6, for ground leaves. The validity of the quadratic models was confirmed by performing extractions under these optimized conditions. The resulting discrepancies between predicted and observed data were minimal, with percentage deviations ranging from 0.04% to 10.2%.

The results obtained showed that the application of UAE for *Rosa canina* L. whole leaves is more effective than SLE. In particular, the use of UAE reduced the duration of the extraction process by 22% while increasing the TPC recovery yield by 31%. This effect indicates an intensification of mass transfer and a more efficient extractability of phenolics from the plant matrix under ultrasonic exposure.

These findings are in good agreement with the extensive literature confirming that UAE is a highly effective method for intensifying the recovery of phenolic compounds and antioxidants from plant matrices. The main mechanism driving this efficiency is acoustic cavitation, which triggers microbubble formation and collapse, facilitating the localized destruction of cell walls, enhancing solvent penetration into cellular structures, and promoting enhanced solvent–matrix interaction and accelerated mass transfer. Numerous studies have emphasized that optimizing UAE operational parameters, including extraction time, ultrasound power, and solid-to-liquid ratio, is essential for maximizing the recovery yield of phenolic compounds [1,30,31]. The efficacy of this technique is consistently demonstrated when comparing UAE to conventional extraction methods for the recovery of BACs from the leaves of different medicinal plants.

González-Silva et al. (2022) [28] optimized the UAE of bioactive compounds from Psidium cattleianum leaves using RSM, testing various extraction times, sonication amplitudes, and pulse cycles. The authors showed that UAE provides a significant increase (15.81%) in the recovery yield of polyphenols (158.18 mg GAE/g_DW_) and antioxidant activity of extracts while reducing total processing time by 96.66% compared to conventional aqueous–organic extraction (AOE).

A study investigating the UAE of bioactive compounds from Crataegus almaatensis leaves showed a 16% increase in TPC alongside a 40% reduction in ethanol usage compared to SLE, demonstrating the process efficiency and environmental friendliness of the approach [23].

A comparative analysis of different extraction methods used to recover phenolic compounds from Lagenaria siceraria showed that UAE provided a significantly higher TPC yield (32.02 mg GAE/100 mg of raw material) compared to Soxhlet extraction (16.51 mg GAE/100 mg), while reducing the processing time [32].

Moreover, the use of UAE with aqueous extractants allowed us to obtain TPC values of approximately 48.59 mg GAE/g_DW_ in *Rosa canina* L. tea extracts, exceeding the values obtained using conventional methods, including Soxhlet extraction [26].

Integrating UAE with enzymatic pretreatments can further enhance the extraction yields of phenolic compounds and their antioxidant activity by facilitating the release of bound phenolics from *Rosa canina* L. fruits, as evidenced by the strong correlation between TPC, FRAP, and ABTS assays [22].

A comparative analysis of the data revealed that utilizing whole leaves of *Rosa canina* L. is a highly efficient extraction strategy, yielding competitive TPC, TFC, and antioxidant values equivalent to, and in some cases exceeding, those obtained from ground material. This approach offers distinct advantages, primarily by minimizing mechanical pre-processing. Avoiding the grinding of leaves, it prevents the potential oxidation or thermal degradation of sensitive phenolic compounds while simultaneously reducing electrical energy consumption. Moreover, to process the same amount of plant material and obtain a comparable recovery of bioactive compounds, ground leaves required a higher amount of solvent (~45%) and a higher concentration of ethanol (70% for ground leaves vs. 40.8% for whole leaves).

The literature indicates that the effect of particle size reduction of plant raw materials on extraction efficiency is ambiguous. While grinding can increase the specific contact surface, thereby enhancing the diffusion of target compounds, it often necessitates a lower solid-to-solvent ratio for effective mass transfer and solubilization of intracellular compounds. This results in a higher total volume of extracting medium, leading to increased consumption of organic solvents [30,33].

Several studies have indicated that increasing the volume of solvents during the extraction of finely dispersed raw materials is accompanied by technological and economic limitations. In particular, the phase separation stages, as well as the processes of concentration and solvent removal, become more complicated, leading to increased energy consumption and reduced overall technological sustainability [30,32]. These aspects highlight the advantages of using whole leaves coupled with intensified extraction methods such as UAE.

These findings confirm that UAE is an efficient and sustainable strategy for the recovery of phenolic compounds from *Rosa canina* L. leaves. Such efficacy justifies its use as an optimal extraction method, paving the way for sustainable, multi-step cascade processing that ensures the full valorization of residual plant biomass.

### 3.3. Cascade Valorization of Residual Biomass via High-Pressure Homogenization

Residual plant material often retains significant quantities of BACs, which remain entrapped within robust cell structures. Such extraction residues are usually disposed of, reducing the overall economic efficiency of the process and contradicting the principles of sustainable development. Therefore, repurposing this residual material for further processing represents a critical step toward the comprehensive valorization of plant-based resources [1].

**Figure 2:**
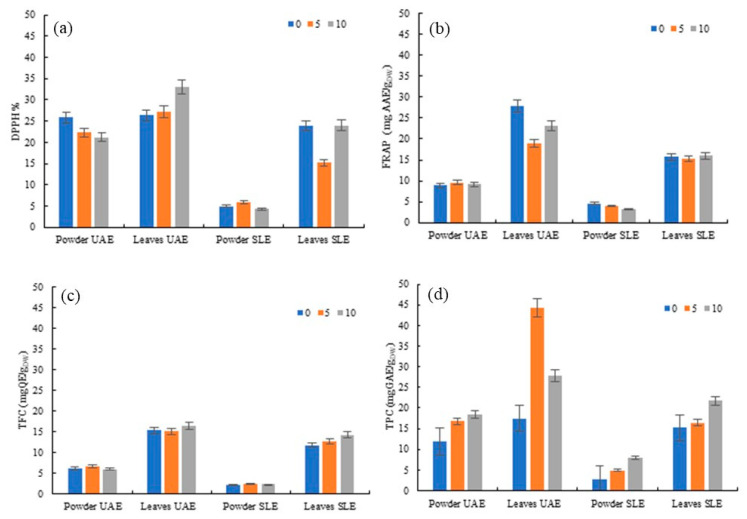
Kinetics of release of antioxidant activity (DPPH (**a**), FRAP (**b**)), total flavonoid content (TFC (**c**)), and total phenolic content (TPC (**d**)) during HPH (0–10 min) from the residual biomass of whole and ground leaves of *Rosa canina* L. after UAE and SLE treatment.

In this regard, HPH was applied after both SLE and UAE processes to evaluate the efficiency of residual bioactive compound recovery. Prior to HPH, samples were subjected to HSM. Subsequently, HPH was performed with sampling at 0, 5, and 10 min. Figure 2 shows the kinetics of BAC release during HPH from the residual biomass of whole and ground leaves of *Rosa canina* L. after UAE and SLE treatment.

A comparative analysis of HPH applied after UAE and SLE showed a clear dependence of extraction yields on the type of biomass and the method applied during the first extraction step. The application of HPH as a post-extraction step serves as an effective strategy for recovering residual bioactive compounds, effectively overcoming the limitations of preliminary SLE and UAE processes. HPH provides mechanical destruction of cell structures due to intense shear stresses, turbulence, and cavitation effects, which increases the permeability of the plant matrix and improves solvent access to intracellular phenolic compounds [1,17].

Extracts from the residual biomass obtained after UAE consistently showed higher levels of bioactive compounds (TFC and TPC) and antioxidant activity (DPPH and FRAP) compared to those obtained after SLE. This suggests that while UAE is already an effective primary extraction method, it better conditions the cell walls for subsequent HPH-mediated release of intracellular compounds, making bioactive compounds more accessible. The combination of UAE and HPH led to the highest content of phenolic compounds and antioxidant activity from the residual biomass of *Rosa canina* L. whole leaves. DPPH activity reached 32.97% after 10 min of treatment, and FRAP values ranged from 19.04 to 27.81 mg AAE/g_DW_. TPC and TFC reached concentrations of 44.37 mg GAE/g_DW_ and 16.45 mg QE/g_DW_, respectively. In contrast, extracts obtained from residues remaining after SLE contained significantly lower concentrations of extractable compounds: TPC (15.20–21.74 mg GAE/g_DW_), TFC (11.78–14.29 mg QE/g_DW_), and antioxidant activity (DPPH 15.20–23.99%; FRAP 15.25–15.98 mg AAE/g_DW_).

Extracts from the residual biomass of ground leaves obtained after UAE and SLE showed significantly lower recovery yields compared to those from whole leaves. Extracts obtained after the cascade process SLE + HPH showed minimal DPPH values (4.28–5.93%), low FRAP values (3.26–4.55 mg AAE/g_DW_), TPC (2.83–8.00 mg GAE/g_DW_), and TFC (2.21–2.39 mg QE/g_DW_).

These results indicate that whole-leaf biomass responds more favourably to treatment compared to ground leaves, likely due to a tissue structure that facilitates greater mechanical disruption during HPH, leading to a more pronounced release of intracellular compounds. This suggests that the energy-intensive grinding stage can be avoided without compromising the extraction yield of BACs. Notably, leaves pre-treated with UAE showed significant enhancements, as evidenced by TPC, which recorded a 158% increase after 5 min of HPH treatment, and a 27% increase in antioxidant capacity (DPPH) after 10 min. TFC further confirms this positive trend with increases of up to 27% in extracts from leaves subjected to SLE. However, the antioxidant capacity showed more fluctuation. In some cases, a slight decrease after 5 or 10 min of HPH was detected. This could indicate the degradation or oxidation of sensitive antioxidant compounds due to the HPH process over extended treatment times.

Literature data confirm that the use of HPH as a preliminary or subsequent processing stage contributes to an increase in the recovery yield of phenolic compounds and antioxidant activity compared to traditional extraction methods. Zhu et al. (2016) [34] showed that the combination of alkaline treatment with HPH used to process potato peels led to a significant increase in the release of phenolic compounds, which was associated with more efficient destruction of cell walls and intensification of mass transfer. Similar effects have been described by Peng et al. (2025) [35], who found that HPH treatment contributed to changing the profile of phenolic compounds in peach juice, increased the antioxidant activity, and improved the bioavailability of phenols during simulated digestion.

Likewise, Yong et al. (2021) [36] emphasized that HPH enhanced both the extractability and stability of phytochemical compounds by reducing particle size and increasing the specific contact area between the plant material and the extractant.

Several studies on the HPH treatment of agri-food residues, such as coffee husks, wheat bran, and tomato pomace [14,17,24], have shown that HPH can increase the yield of phenolic compounds and antioxidant activity by approximately 20% compared to conventional HSM. While these studies did not specifically focus on *Rosa canina* L., the observed increase in mass transfer, driven by mechanical cell disruption, aligns with the findings of the present study.

The results obtained corroborate the synergistic potential of UAE and HPH and the validity of integrating these two technologies within a cascade extraction process. While the initial ultrasonic treatment facilitates the recovery of readily accessible phenolic compounds, the subsequent HPH treatment ensures additional release of bound bioactive fractions from the residual biomass.

## 4. Conclusions

The present study successfully optimized the valorization of *Rosa canina* L. leaves through a comparative assessment of conventional and intensified approaches to recover phenolics, flavonoids, and antioxidants from both whole and ground leaves. The obtained findings demonstrate that ultrasound-assisted extraction (UAE) of whole leaves offers a superior alternative to solid–liquid extraction (SLE), achieving a 31% increase in total phenolic content (TPC) while simultaneously reducing the duration of the extraction process. This confirms the efficiency of ultrasonic cavitation in accelerating mass transfer without the need for prior energy-intensive grinding.

A critical advantage of processing whole leaves lies in the optimization of solvent use and improved downstream feasibility. Unlike ground leaves, which required a lower solid-to-liquid ratio (0.05 g/mL) to ensure adequate diffusion, resulting in 45% higher solvent consumption and a 42% higher ethanol concentration in water, whole leaf extraction operates efficiently with a lower environmental impact. The integration of high-pressure homogenization (HPH) as a subsequent stage further validated this approach. Kinetic analysis revealed that HPH effectively recovered the remaining bioactive fractions (TFC, TPC, DPPH, and FRAP) from the residual biomass, with whole leaves exhibiting a higher degree of release compared to their ground counterparts.

Collectively, these results confirm the high efficiency of the UAE–HPH cascade process, representing a technologically robust and feasible strategy for the sustainable valorization of *Rosa canina* L. leaves.

Future perspectives should focus on the validation of the obtained findings for the scale-up of this integrated UAE–HPH process, evaluating its economic viability and exploring the potential bioactivity of the extracted fractions in real-world applications.

## Figures and Tables

**Table 1:** Experimental design of the SLE input variables and the responses TPC (mg GAE/g_DW_), TFC (mg QE/g_DW_), FRAP (mg AAE/g_DW_), and DPPH (%) for the rosehip whole leaves extracts.

Run	Input				Responses			
	t (min)	T (°C)	EtOH (%)	S/L (g/mL)	TPC	TFC	FRAP	DPPH
1	90	25	0	0.02	44.85	38.43	72.27	57.74
2	90	25	80	0.075	139.44	171.07	121.62	66.8
3	50	25	40	0.0475	44.22	44.94	57.21	63.06
4	10	25	0	0.02	37.99	33.17	90.91	59.68
5	10	25	80	0.02	61.50	4.87	37.62	54.39
6	10	25	80	0.075	11.93	8.92	18.79	34.39
7	90	25	80	0.02	23.03	16.75	56.10	59.29
8	90	25	0	0.075	64.92	85.30	113.05	69.03
9	10	25	0	0.075	25.76	24	37.17	55.66
10	50	47.5	40	0.0475	132.46	138.28	119.66	66.37
11	50	47.5	80	0.0475	59.73	65.11	59.59	38.26
12	10	47.5	40	0.0475	115.30	106.57	106.97	66.8
13	90	47.5	40	0.0475	246.70	194.52	227.67	75
14	50	47.5	0	0.0475	104.73	89.17	128.12	71.01
15	50	47.5	40	0.075	146.11	138.49	263.38	75.43
16	50	47.5	40	0.02	108.65	75.63	127.29	68.72
17	90	70	80	0.075	71.29	96.05	72.60	60.51
18	90	70	0	0.02	208.50	247.03	234.90	80.13
19	10	70	0	0.02	94.70	85.84	128.25	74.78
20	10	70	0	0.075	86.79	98.17	102.94	73.73
21	10	70	80	0.075	77.83	69.06	67.69	54.39
22	90	70	80	0.02	93.43	82.64	137.34	83.46
23	10	70	80	0.02	43.89	35.88	87.24	62.69
24	90	70	0	0.075	145.65	161.6	263.38	81.04
25	50	70	40	0.0475	108.16	133.27	147.82	68.07

**Table 2:** Experimental design of the SLE input variables and the responses TPC (mg GAE/g_DW_), TFC (mg QE/g_DW_), FRAP (mg AAE/g_DW_), DPPH (%) for the rosehip ground leaves extracts.

Run	Input			Responses			
	t (min)	T (°C)	EtOH (%)	TPC	TFC	FRAP	DPPH
1	90	25	0	110.20	65.40	120.50	58.10
2	90	25	80	165.40	110.20	175.60	66.40
3	50	25	40	115.30	75.80	130.40	60.20
4	10	25	0	75.80	45.20	85.30	52.10
5	10	25	80	105.40	60.70	110.20	55.40
6	10	25	80	105.50	60.90	110.50	55.50
7	90	25	80	165.20	110.50	175.40	66.50
8	90	25	0	110.50	65.20	120.30	58.30
9	10	25	0	75.60	45.50	85.10	52.30
10	50	47.5	40	160.40	105.70	185.30	68.40
11	50	47.5	80	195.80	130.40	210.50	72.10
12	10	47.5	40	130.20	85.60	145.20	62.50
13	90	47.5	40	190.50	125.30	215.40	73.60
14	50	47.5	0	135.60	90.20	150.80	64.20
15	50	47.5	40	160.20	105.50	185.10	68.20
16	50	47.5	40	160.50	105.90	185.50	68.60
17	90	70	80	224.10	150.80	250.40	75.90
18	90	70	0	165.30	115.40	180.20	68.10
19	10	70	0	115.40	75.60	125.30	60.40
20	10	70	0	115.20	75.40	125.10	60.20
21	10	70	80	150.60	102.30	165.40	65.80
22	90	70	80	224.50	151.10	250.80	76.05
23	10	70	80	150.20	102.10	165.10	65.60
24	90	70	0	165.80	115.80	180.50	68.30
25	50	70	40	185.40	125.60	210.30	71.40

**Table 3:** Experimental design of the UAE input variables and the responses TPC (mg GAE/g_DW_), TFC (mg QE/g_DW_), FRAP (mg AAE/g_DW_), DPPH (%) for the rosehip whole leaves extracts.

Run	Input			Responses			
	t (min)	T (°C)	EtOH (%)	TPC	TFC	FRAP	DPPH
1	90	40	70	210.51	85.20	150.20	60.10
2	30	40	20	185.62	75.80	165.40	62.30
3	90	40	20	225.33	115.60	195.80	68.40
4	30	40	70	170.85	55.50	130.50	56.20
5	60	40	45	285.30	165.90	260.40	74.10
6	60	55	45	295.80	175.40	282.15	78.50
7	90	55	45	292.40	172.20	280.50	77.90
8	60	55	20	225.74	115.30	210.30	70.20
9	60	55	70	230.17	108.70	185.60	65.40
10	30	55	45	240.56	130.10	220.40	68.80
11	60	70	45	265.25	140.50	230.10	69.50
12	30	70	20	155.94	67.20	140.20	58.10
13	90	70	70	195.43	72.60	145.30	59.20
14	90	70	20	180.27	70.10	170.50	62.40
15	30	70	70	145.51	50.80	115.80	54.30

**Table 4:** Experimental design of the UAE input variables and the responses TPC (mg GAE/g_DW_), TFC (mg QE/g_DW_), FRAP (mg AAE/g_DW_), DPPH (%) for the rosehip ground leaves extracts.

Run	Input			Responses			
	t (min)	T (°C)	EtOH (%)	TPC	TFC	FRAP	DPPH
1	90	40	70	195.40	115.20	210.50	65.20
2	30	40	20	145.30	90.70	150.30	54.50
3	90	40	20	160.90	98.60	170.20	58.20
4	30	40	70	175.20	102.70	185.60	60.80
5	60	40	45	185.90	115.30	195.40	63.15
6	60	55	45	210.90	128.20	240.80	68.50
7	90	55	45	225.40	132.60	260.40	70.30
8	60	55	20	180.80	108.30	205.10	62.40
9	60	55	70	230.60	135.30	270.20	71.20
10	30	55	45	190.80	118.70	215.60	65.80
11	60	70	45	238.15	138.50	282.35	73.90
12	30	70	20	185.80	110.10	205.10	61.60
13	90	70	70	245.30	140.80	285.80	74.10
14	90	70	20	195.50	118.30	220.20	64.10
15	30	70	70	225.20	130.40	260.90	70.50

**Table 5:** Total phenolic content (TPC), total flavonoid content (TFC), and antioxidant activity (FRAP and DPPH) of extracts from *Rosa canina* L. whole leaves obtained after SLE and UAE processes under optimized conditions.

	SLE		UAE	
**Optimal parameters**	T: 55.6 °C t: 90 min EtOH: 32.4% S/L: 0.072 g/mL		T: 55.5 °C t: 69.7 min EtOH: 40.8% S/L: 0.072 g/mL US power: 100 W	
	**Predicted**	**Experimental**	**Predicted**	**Experimental**
TPC (mg GAE/g_DW_)	216.92	220.70 ± 0.20	289.11	289.55 ± 0.85
TFC (mg QE/g_DW_)	203.66	194.72 ± 0.80	174.52	177.88 ± 0.77
FRAP (mg AAE/g_DW_)	263.39	247.65 ± 1.30	285.98	284.9 ± 1.15
DPPH (%)	83.55	75 ± 1.50	79.54	76.85 ± 1.10

**Table 6:** TPC, TFC, FRAP, and DPPH values of extracts from *Rosa canina* L. ground leaves obtained after SLE and UAE processes under optimized conditions.

	SLE		UAE	
**Optimal parameters**	T: 69 °C t: 88 min EtOH: 78% S/L: 0.05 g/mL		T: 68.2 °C t: 60 min EtOH: 68% S/L: 0.05 g/mL US power: 100 W	
	**Predicted**	**Experimental**	**Predicted**	**Experimental**
TPC (mg GAE/g_DW_)	225.40	225.50 ± 1.10	249.67	245.50 ± 0.90
TFC (mg QE/g_DW_)	152.15	150.98 ± 0.95	142.60	140.78 ± 0.65
FRAP (mg AAE/g_DW_)	251.30	255.03 ± 1.80	292.05	289.2 ± 1.50
DPPH (%)	76.10	75.07 ± 1.25	74.90	73.87 ± 0.95

## Data Availability

The data presented in this study are available within the article. The original data that support the findings of this study are available from the corresponding authors, upon reasonable request.

## Supplementary Materials

The following supporting information can be downloaded at https://www.mdpi.com/article/10.3390/antiox15050560/s1, Figure S1: 3D response surface graphs for TPC (mg GAE/g_DW_) (a,c,e) and TFC (mg QE/g_DW_) (b,d,f) of rosehip whole leaves extracts after SLE as a function of temperature and time, at a S/L ratio of 0.072 g/mL, and at ethanol concentrations in water (*v*/*v*) of 0% (a,b), 40% (c,d), and 80% (e,f); Figure S2: 3D response surface graphs for FRAP (mg AAE/g_DW_) (a,c,e) and DPPH (%) (b,d,f) of rosehip whole leaves extracts after SLE as a function of temperature and time, at a S/L ratio of 0.072 g/mL, and at ethanol concentrations in water (*v*/*v*) of 0% (a,b), 40% (c,d), and 80% (e,f); Figure S3: Predicted vs. actual values of TPC (a), TFC (b), FRAP (c), and DPPH (d) of rosehip whole leaves extracts obtained after SLE; Figure S4: Normal plots of residuals for TPC (a), TFC (b), FRAP (c), and DPPH (d) of rosehip whole leaves extracts obtained after SLE; Figure S5: 3D response surface graphs for TPC (mg GAE/g_DW_) (a,c,e) and TFC (mg QE/g_DW_) (b,d,f) of rosehip whole leaves extracts after UAE as a function of temperature and time, at S/L ratio of 0.072 g/mL, and at ethanol concentrations in water (*v*/*v*) of 20% (a,b), 45% (c,d), and 70% (e,f); Figure S6: 3D response surface graphs for FRAP (mg AAE/g_DW_) (a,c,e) and DPPH (%) (b,d,f) of rosehip whole leaves extracts after UAE as a function of temperature and time, at a S/L ratio of 0.072 g/mL, and at ethanol concentrations in water (*v*/*v*) of 20% (a,b), 45% (c,d), and 70% (e,f); Figure S7: Predicted vs. actual values of TPC (a), TFC (b), FRAP (c), and DPPH (d) of rosehip whole leaves extracts obtained after UAE; Figure S8: Normal plots of residuals for TPC (a), TFC (b), FRAP (c), and DPPH (d) of rosehip whole leaves extracts obtained after UAE; Figure S9: 3D response surface graphs for TPC (mg GAE/g_DW_) (a,c,e) and TFC (mg QE/g_DW_) (b,d,f) of rosehip ground leaves extracts after SLE as a function of temperature and time, at a S/L ratio of 0.05 g/mL, and at ethanol concentrations in water (*v*/*v*) of 0% (a,b), 40% (c,d), and 80% (e,f); Figure S10: 3D response surface graphs for FRAP (mg AAE/g_DW_) (a,c,e) and DPPH (%) (b,d,f) of rosehip ground leaves extracts after SLE as a function of temperature and time, at a S/L ratio of 0.05 g/mL, and at ethanol concentrations in water (*v*/*v*) of 0% (a,b), 40% (c,d), and 80% (e,f); Figure S11: Predicted vs. actual values of TPC (a), TFC (b), FRAP (c), and DPPH (d) of rosehip ground leaves extracts obtained after SLE; Figure S12: Normal plots of residuals for TPC (a), TFC (b), FRAP (c), and DPPH (d) of rosehip ground leaves extracts obtained after SLE; Figure S13: 3D response surface graphs for TPC (mg GAE/g_DW_) (a,c,e) and TFC (mg QE/g_DW_) (b,d,f) of rosehip ground leaves extracts after UAE as a function of temperature and time, at a S/L ratio of 0.05 g/mL, and at ethanol concentrations in water (*v*/*v*) of 20% (a,b), 45% (c,d), and 70% (e,f); Figure S14: 3D response surface graphs for FRAP (mg AAE/g_DW_) (a,c,e) and DPPH (%) (b,d,f) of rosehip ground leaves extracts after UAE as a function of temperature and time, at a S/L ratio of 0.05 g/mL, and at ethanol concentrations in water (*v*/*v*) of 20% (a,b), 45% (c,d), and 70% (e,f); Figure S15: Predicted vs. actual values of TPC (a), TFC (b), FRAP (c), and DPPH (d) of rosehip ground leaves extracts obtained after UAE; Figure S16: Normal plots of residuals for TPC (a), TFC (b), FRAP (c), and DPPH (d) of rosehip ground leaves extracts obtained after UAE; Table S1: Second-order polynomial model coefficients for TPC, TFC, FRAP, and DPPH of rosehip whole leaves extracts obtained after SLE; Table S2: Second-order polynomial model coefficients for TPC, TFC, FRAP, and DPPH of rosehip whole leaves extracts obtained after UAE; Table S3: Second-order polynomial model coefficients for TPC, TFC, FRAP, and DPPH of rosehip ground leaves extracts obtained after SLE; Table S4: Second-order polynomial model coefficients for TPC, TFC, FRAP, and DPPH of rosehip ground leaves extracts obtained after UAE.
