# Supplementary material for: Enhanced Recovery of Bioactive Compounds from Rosa canina L. Leaves: A Cascade Approach Using Ultrasounds and High-Pressure Homogenization
Source: Antioxidants (Basel). 2026 Apr 28;15(5):560. doi: 10.3390/antiox15050560 (PMC13203715; doi:10.3390/antiox15050560)
Supplement: Supplementary file 1 [file antioxidants-15-00560-s001.zip › antioxidants-4247981-supplementary.pdf]

Supplementary material

Table S1. Second-order polynomial model coefficients for TPC, TFC, FRAP, and DPPH of rosehip whole leaves extracts obtained after SLE.

**Coefficients**								
	TPC (mgGAE/g_{DW})		TFC (mgQE/g_{DW})		FRAP (mgAAE/g_{DW})		DPPH	
α_0	-205.616	*	-181.360	*	-91.582	**	55.260	***
α_1 (time)	-2.879	**	-1.6358	**	-0.938	**	-0.266	**
α_2 (T)	12.515	**	8.313	**	11.420	**	0.653	**
α_3 (EtOH)	2.885	ns	2.0448	ns	3.425	*	0.561	**
α_4 (S/L ratio)	517.05	ns	1892.049	ns	-5373.271	ns	-626.777	ns
α_{12} (t x T)	0.00559	ns	0.00401	ns	0.00997	ns	-0.000635	ns
α_{13} (t x EtOH)	-0.00338	ns	-0.00169	ns	-0.00578	ns	0.00156	ns
α_{14} (t x S/L)	4.958	ns	5.0432	ns	10.652	ns	1.716	ns
α_{23} (T x EtOH)	-0.0216	*	-0.0229	*	-0.0198	ns	-0.00148	ns
α_{24} (T x S/L)	-13.5006	ns	-22.4837	ns	-11.599	ns	-2.650	ns
α_{34} (EtOH x S/L)	8.0429	ns	13.657	ns	-1.581	ns	-2.886	ns
α_{11} (t x t)	0.0318	*	0.0214	ns	0.0105	ns	0.00286	ns
α_{22} (T x T)	-0.106	*	-0.0536	ns	-0.0947	ns	-0.00151	ns
α_{33} (EtOH x EtOH)	-0.0299	ns	-0.0244	ns	-0.0354	ns	-0.00729	*
α_{44} (S/L x S/L)	-3556.527	ns	-12137.213	ns	59305.571	ns	7599.537	ns
p value of the model	0.038	*	0.02	*	0.038	*	0.031	*
R^2	0.894		0.840		0.884		0.893	
Adjusted R^2	0.852		0.801		0.845		0.850	
Predicted R^2	0.781		0.715		0.762		0.778	
Lack of fit	F value: 0.072	ns	F value: 0.280	ns	F value: 0.085	ns	F value: 0.190	ns

ns not significant for $p > 0.05$

*Significant for $p \leq 0.05$; **significant for $p \leq 0.01$; ***significant for $p \leq 0.001$

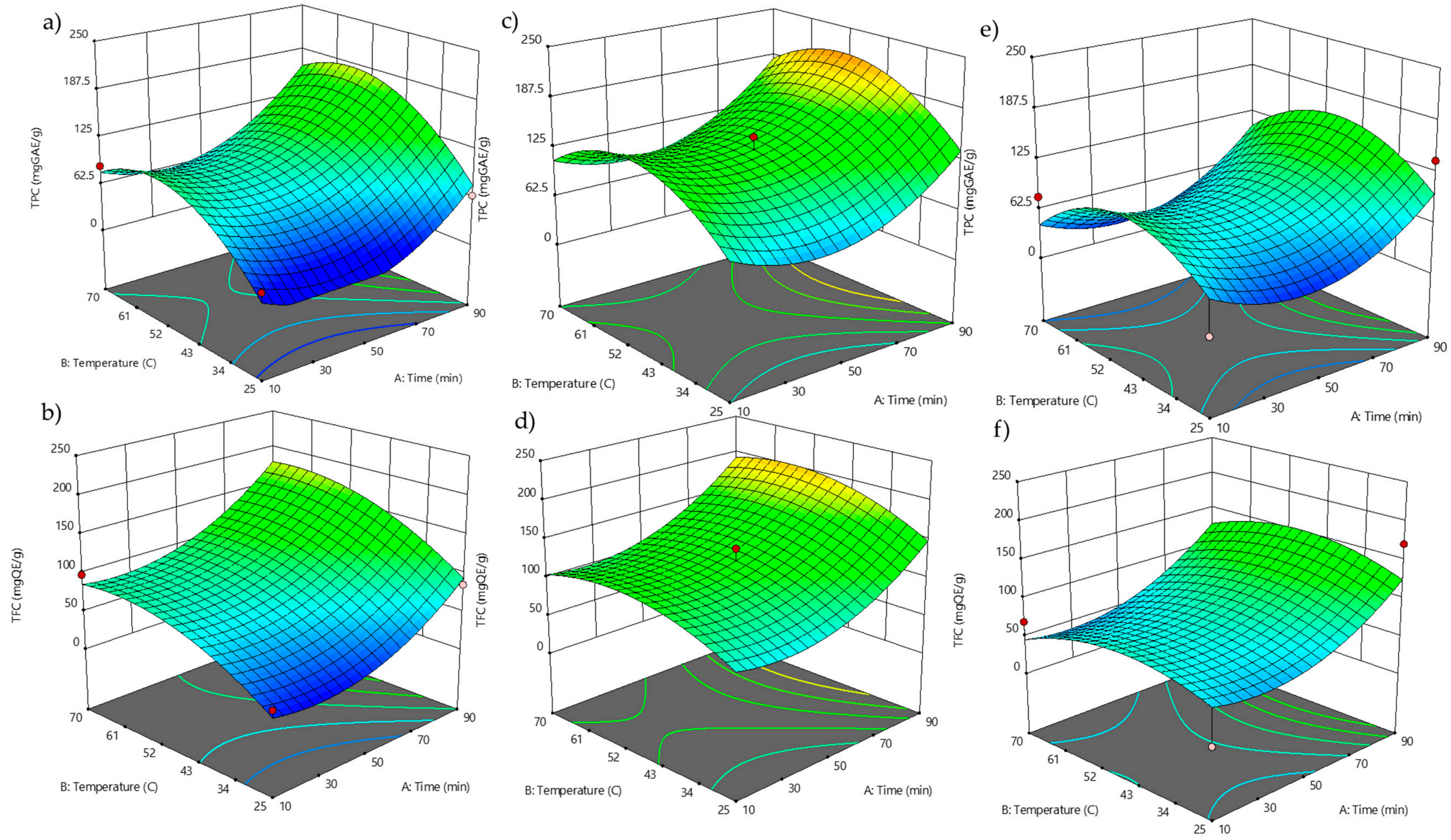

Figure S1. 3D response surface graphs for TPC (mg GAE/g_{DW}) (a, c, e) and TFC (mg QE/g_{DW}) (b, d, f) of rosehip whole leaves extracts after SLE as a function of temperature and time, at a S/L ratio of 0.072 g/ml, and at ethanol concentrations in water (v/v) of 0% (a, b), 40% (c, d), and 80% (e, f).

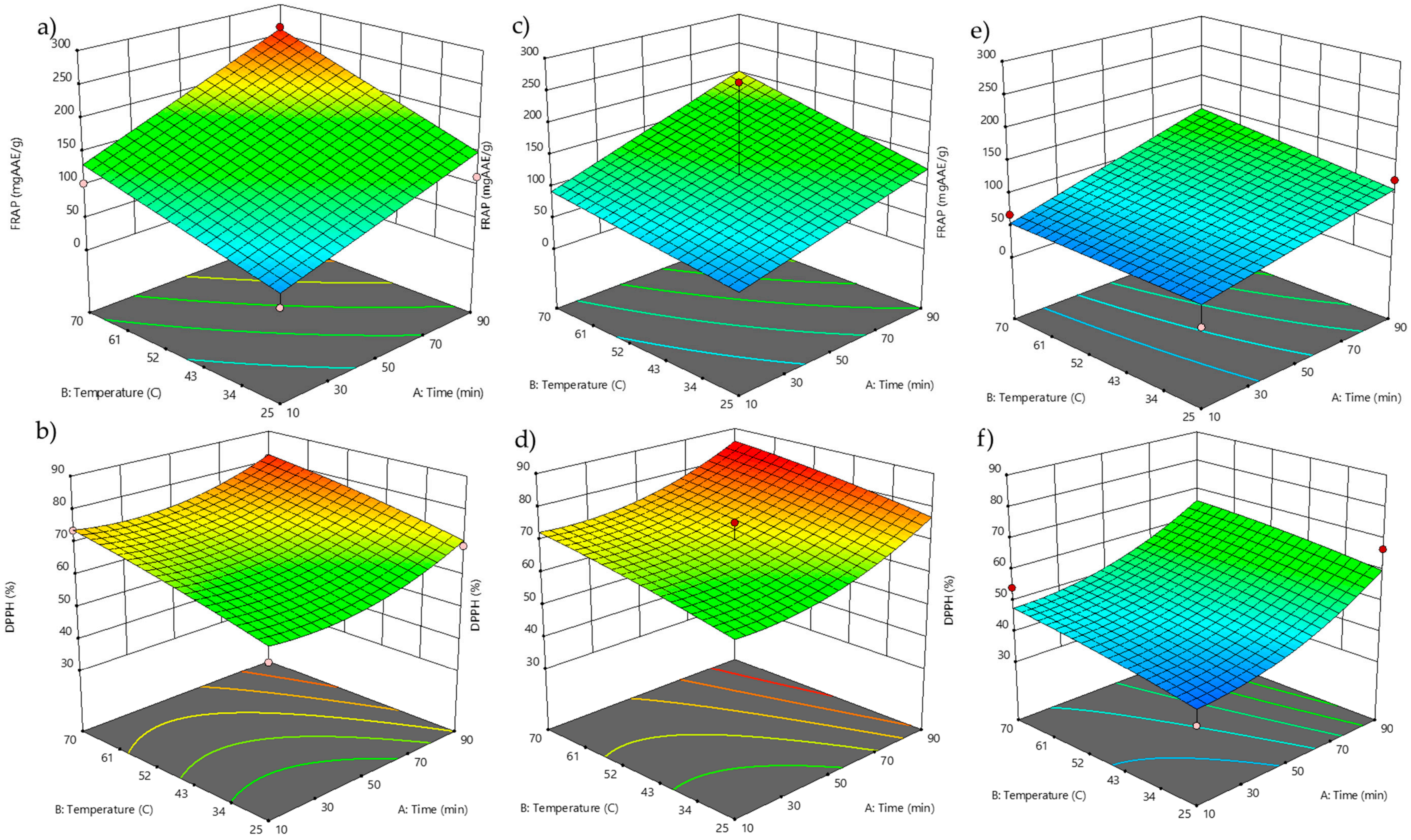

Figure S2. 3D response surface graphs for FRAP (mg AAE/g_{DW}) (a, c, e) and DPPH (%) (b, d, f) of rosehip whole leaves extracts after SLE as a function of temperature and time, at a S/L ratio of 0.072 g/ml, and at ethanol concentrations in water (v/v) of 0% (a, b), 40% (c, d), and 80% (e, f).

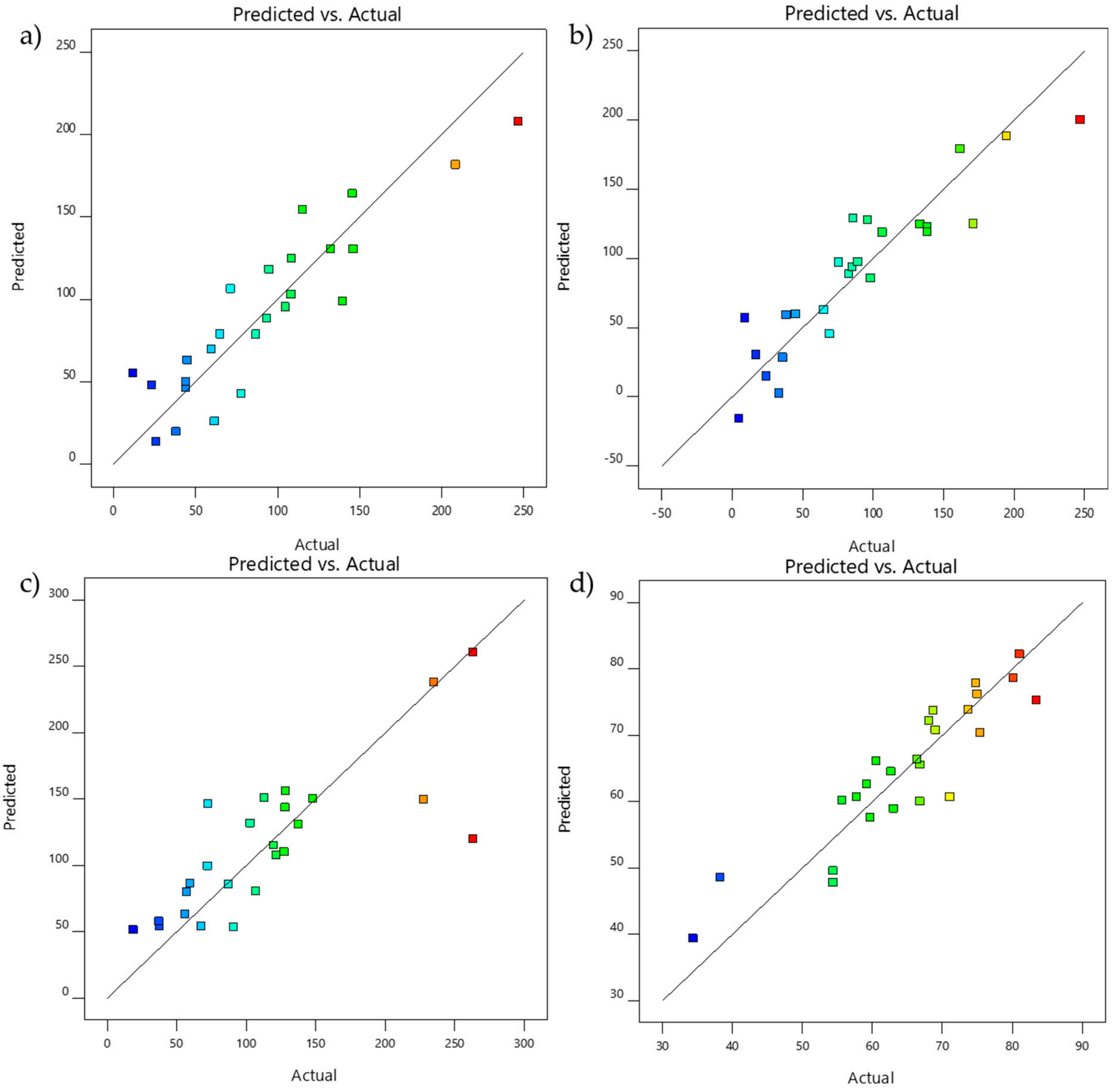

Figure S3. Predicted vs. actual values of TPC (a), TFC (b), FRAP (c), and DPPH (d) of rosehip whole leaves extracts obtained after SLE.

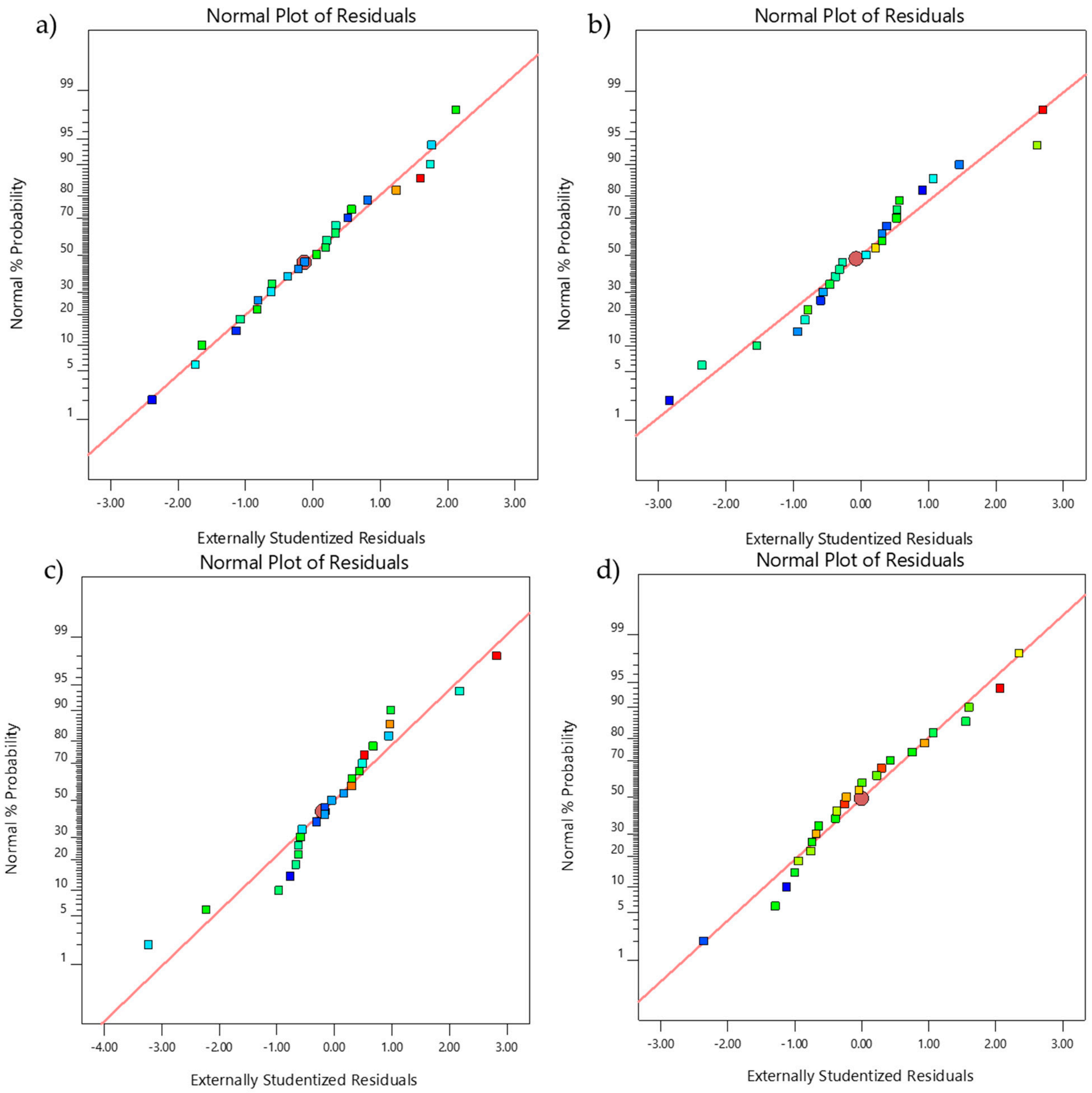

Figure S4. Normal plots of residuals for TPC (a), TFC (b), FRAP (c), and DPPH (d) of rosehip whole leaves extracts obtained after SLE.

Table S2. Second-order polynomial model coefficients for TPC, TFC, FRAP, and DPPH of rosehip whole leaves extracts obtained after UAE.

**Coefficients**								
	TPC (mgGAE/g_{DW})		TFC (mgQE/g_{DW})		FRAP (mgAAE/g_{DW})		DPPH	
α_0	-220.722	*	-498.721	*	-320.848	**	49.500	***
α_1 (time)	4.15065	***	4.506	**	4.0784	**	0.537	**
α_2 (T)	7.493	**	11.661	ns	9.387	*	0.545	***
α_3 (EtOH)	8.447	ns	9.0153	ns	7.851	ns	-0.272	***
α_{12} (t x T)	-0.00142	ns	-0.00217	ns	-0.000889	ns	0.00117	ns
α_{13} (t x EtOH)	0.00426	ns	0.00430	ns	0.00393	ns	0.00146	ns
α_{23} (T x EtOH)	0.0114	ns	-6.667e-05	ns	0.00213	ns	0.00580	**
α_{11} (t x t)	-0.0298	**	-0.0357	***	-0.0324	**	-0.00506	**
α_{22} (T x T)	-0.0802	**	-0.107	**	-0.0896	**	-0.00968	*
α_{33} (EtOH x EtOH)	-0.104	***	-0.105	***	-0.0925	***	-0.00276	ns
p value of the model	< 0.0001	***	0.0002	***	0.0003	*	0.001	***
R^2	0.993		0.990		0.989		0.978	
Adjusted R^2	0.980		0.966		0.958		0.957	
Predicted R^2	0.900		0.830		0.872		0.867	
Lack of fit	F value: 0.180	ns	F value: 0.201	ns	F value: 0.170	ns	F value: 0.075	ns

ns not significant for $p > 0.05$

*Significant for $p \leq 0.05$; **significant for $p \leq 0.01$; ***significant for $p \leq 0.001$

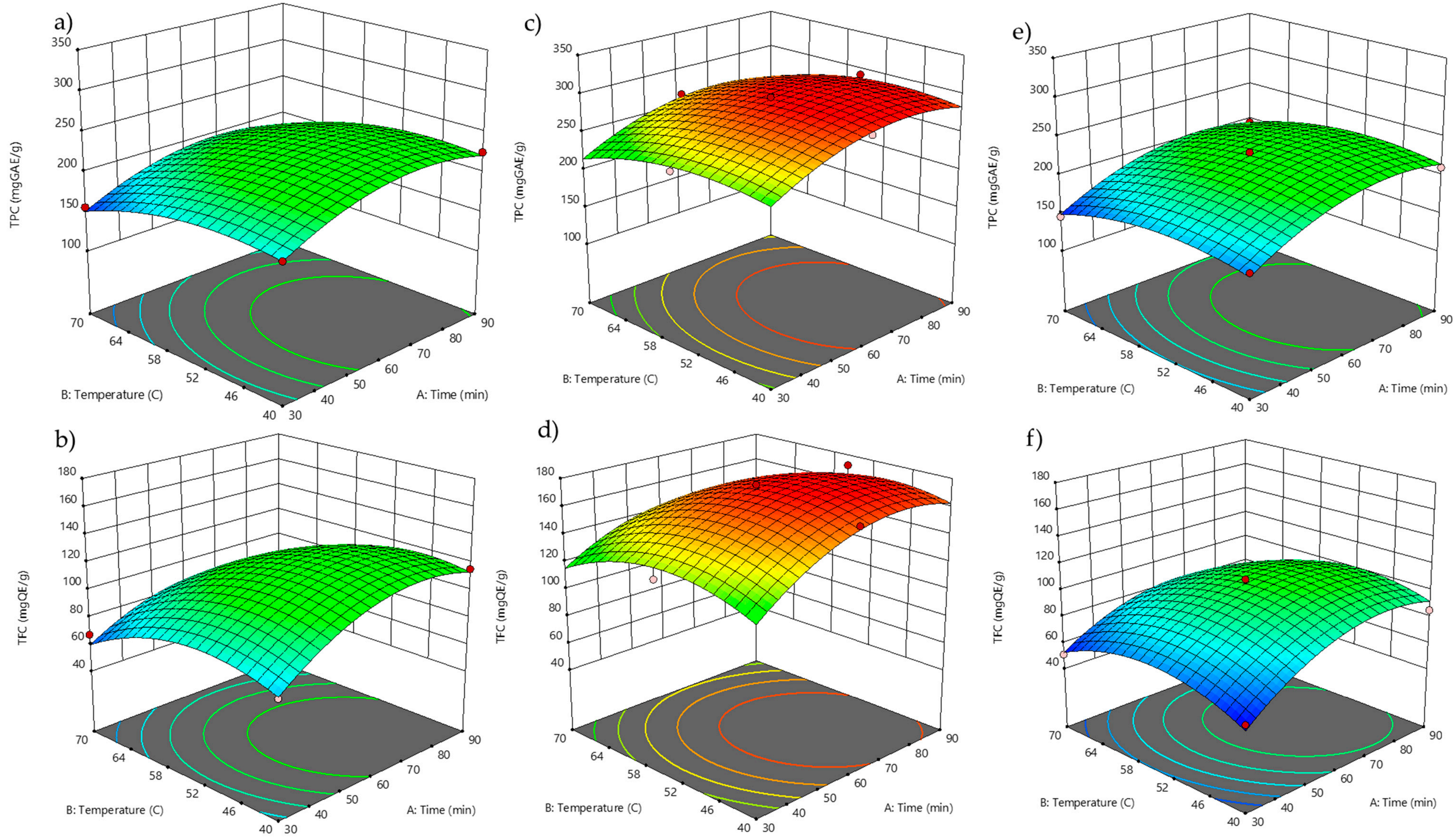

Figure S5. 3D response surface graphs for TPC (mg GAE/g_{DW}) (a, c, e) and TFC (mg QE/g_{DW}) (b, d, f) of rosehip whole leaves extracts after UAE as a function of temperature and time, at S/L ratio of 0.072 g/ml, and at ethanol concentrations in water (v/v) of 20% (a, b), 45% (c, d), and 70% (e, f).

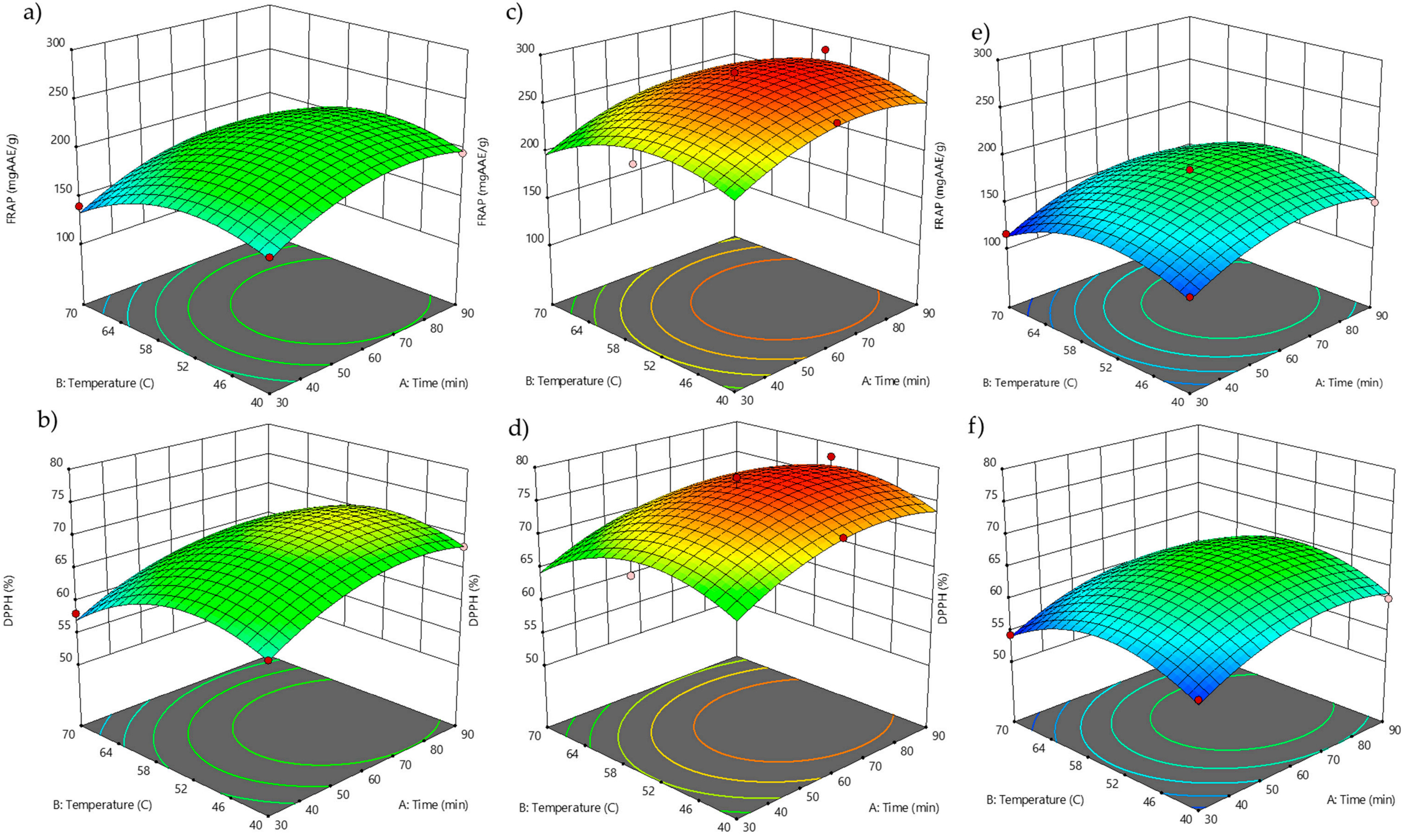

Figure S6. 3D response surface graphs for FRAP (mg AAE/gDW) (a, c, e) and DPPH (%) (b, d, f) of rosehip whole leaves extracts after UAE as a function of temperature and time, at a S/L ratio of 0.072 g/ml, and at ethanol concentrations in water (v/v) of 20% (a, b), 45% (c, d), and 70% (e, f).

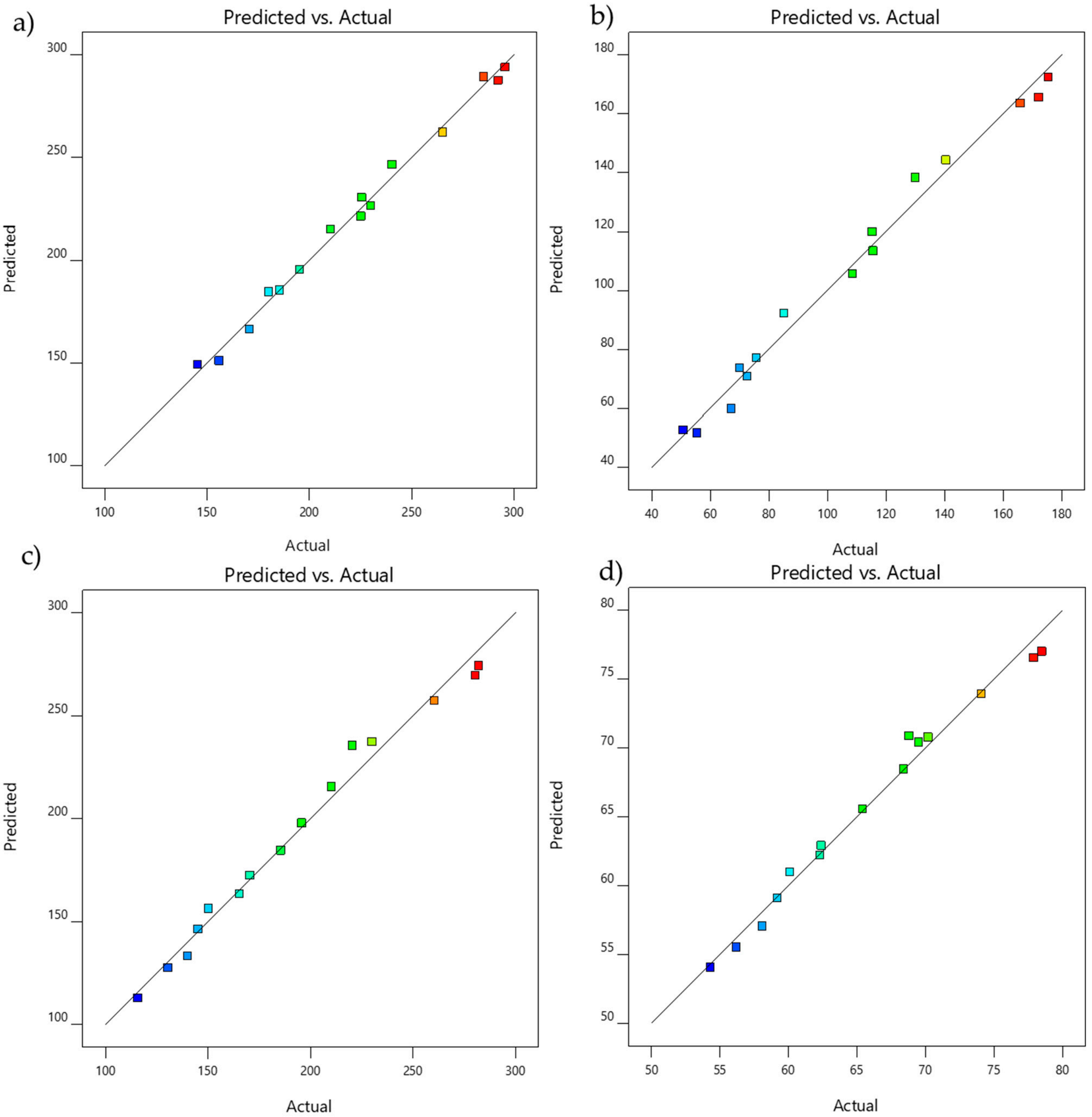

Figure S7. Predicted vs. actual values of TPC (a), TFC (b), FRAP (c), and DPPH (d) of rosehip whole leaves extracts obtained after UAE.

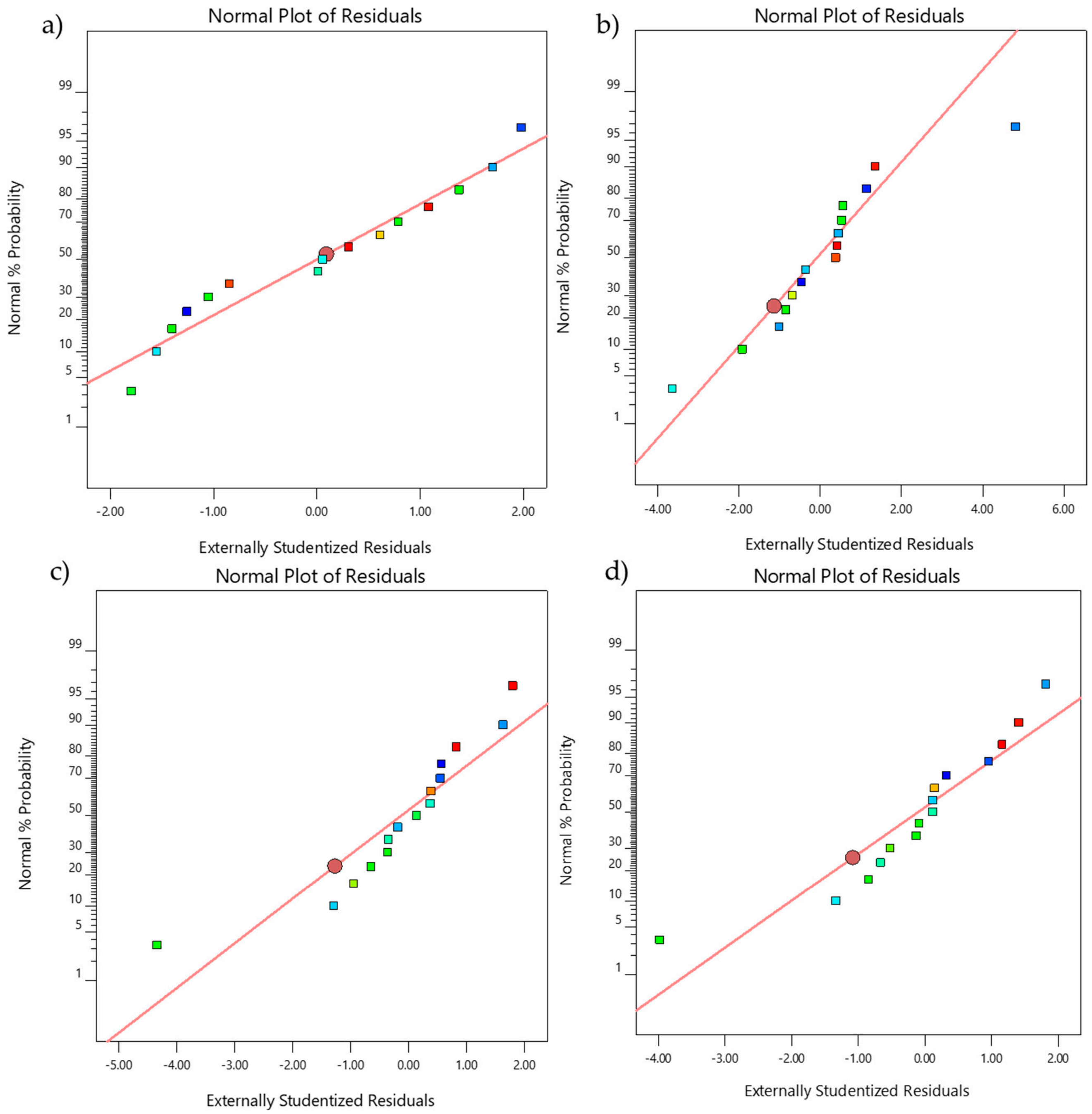

Figure S8. Normal plots of residuals for TPC (a), TFC (b), FRAP (c), and DPPH (d) of rosehip whole leaves extracts obtained after UAE.

Table S3. Second-order polynomial model coefficients for TPC, TFC, FRAP, and DPPH of rosehip ground leaves extracts obtained after SLE.

**Coefficients**								
	TPC ($mgGAE/g_{DW}$)		TFC ($mgQE/g_{DW}$)		FRAP ($mgAAE/g_{DW}$)		DPPH	
α_0	-14.903	*	-21.283	*	-12.967	*	32.753	**
α_1 (time)	0.762	***	0.623	***	0.855	***	0.164	***
α_2 (T)	4.0342	***	2.801	***	4.380	***	0.866	***
α_3 (EtOH)	0.397	***	0.294	***	0.5947	***	0.100	***
α_{12} (t x T)	0.00412	**	0.00269	*	0.00558	***	0.000163	ns
α_{13} (t x EtOH)	0.00382	***	0.00299	***	0.00470	***	0.000576	***
α_{23} (T x EtOH)	0.00127	ns	0.000215	ns	0.00417	**	0.000233	ns
α_{11} (t x t)	-0.00420	*	-0.00376	*	-0.00541	**	-0.000821	**
α_{22} (T x T)	-0.0330	***	-0.0213	***	-0.0368	***	-0.00704	***
α_{33} (EtOH x EtOH)	-0.000852	ns	-0.000724	ns	-0.00520	**	-0.000758	*
p value of the model	< 0.0001	***	< 0.0001	***	< 0.0001	***	< 0.0001	***
R^2	0.988		0.988		0.993		0.992	
Adjusted R^2	0.984		0.981		0.989		0.988	
Predicted R^2	0.967		0.964		0.978		0.975	
Lack of fit	F value: 0.250	ns	F value: 0.112	ns	F value: 0.111	ns	F value: 0.210	ns

ns not significant for $p > 0.05$

*Significant for $p \leq 0.05$; **significant for $p \leq 0.01$; ***significant for $p \leq 0.001$

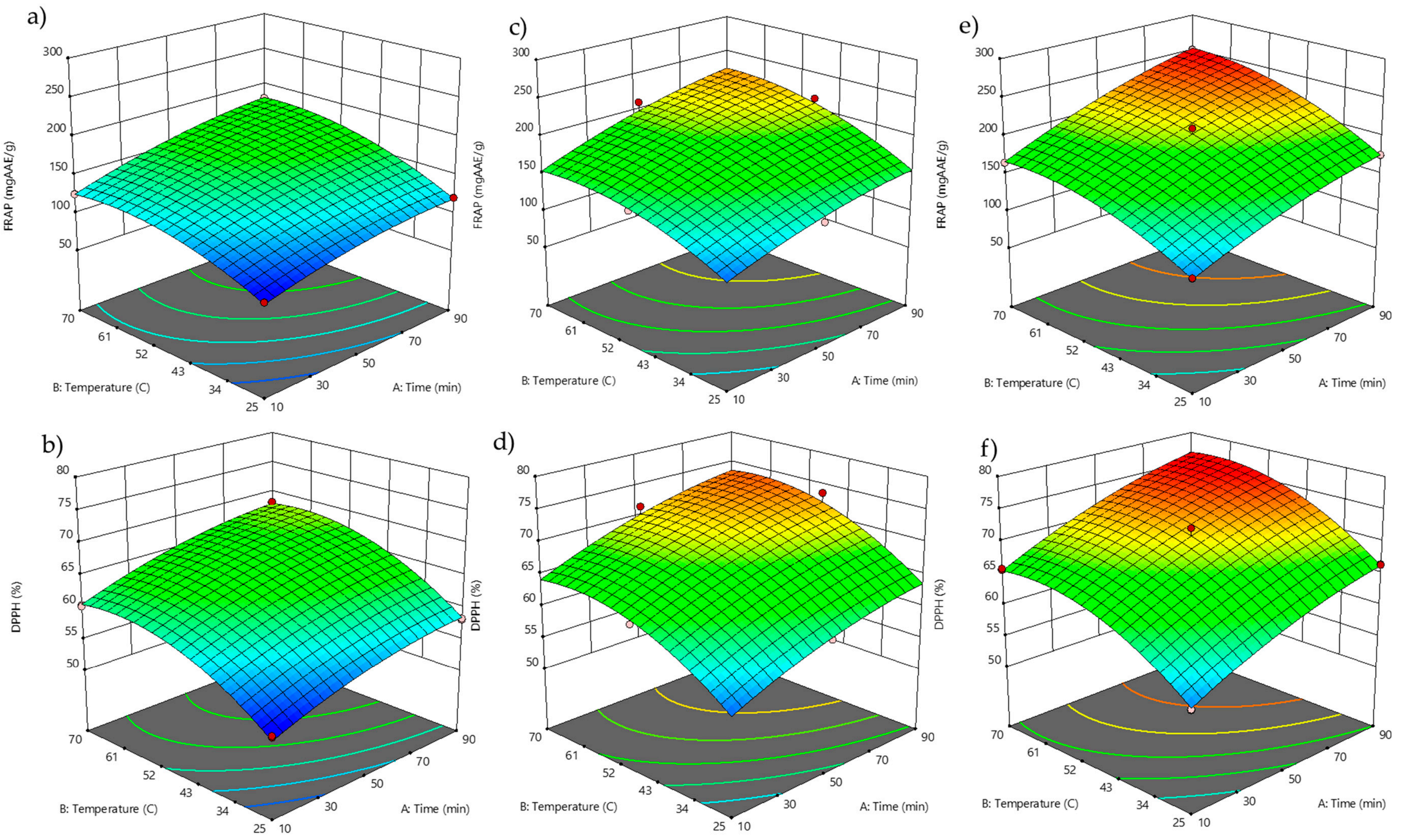

Figure S9. 3D response surface graphs for TPC (mg GAE/g_{DW}) (a, c, e) and TFC (mg QE/g_{DW}) (b, d, f) of rosehip ground leaves extracts after SLE as a function of temperature and time, at a S/L ratio of 0.05 g/ml, and at ethanol concentrations in water (v/v) of 0% (a, b), 40% (c, d), and 80% (e, f).

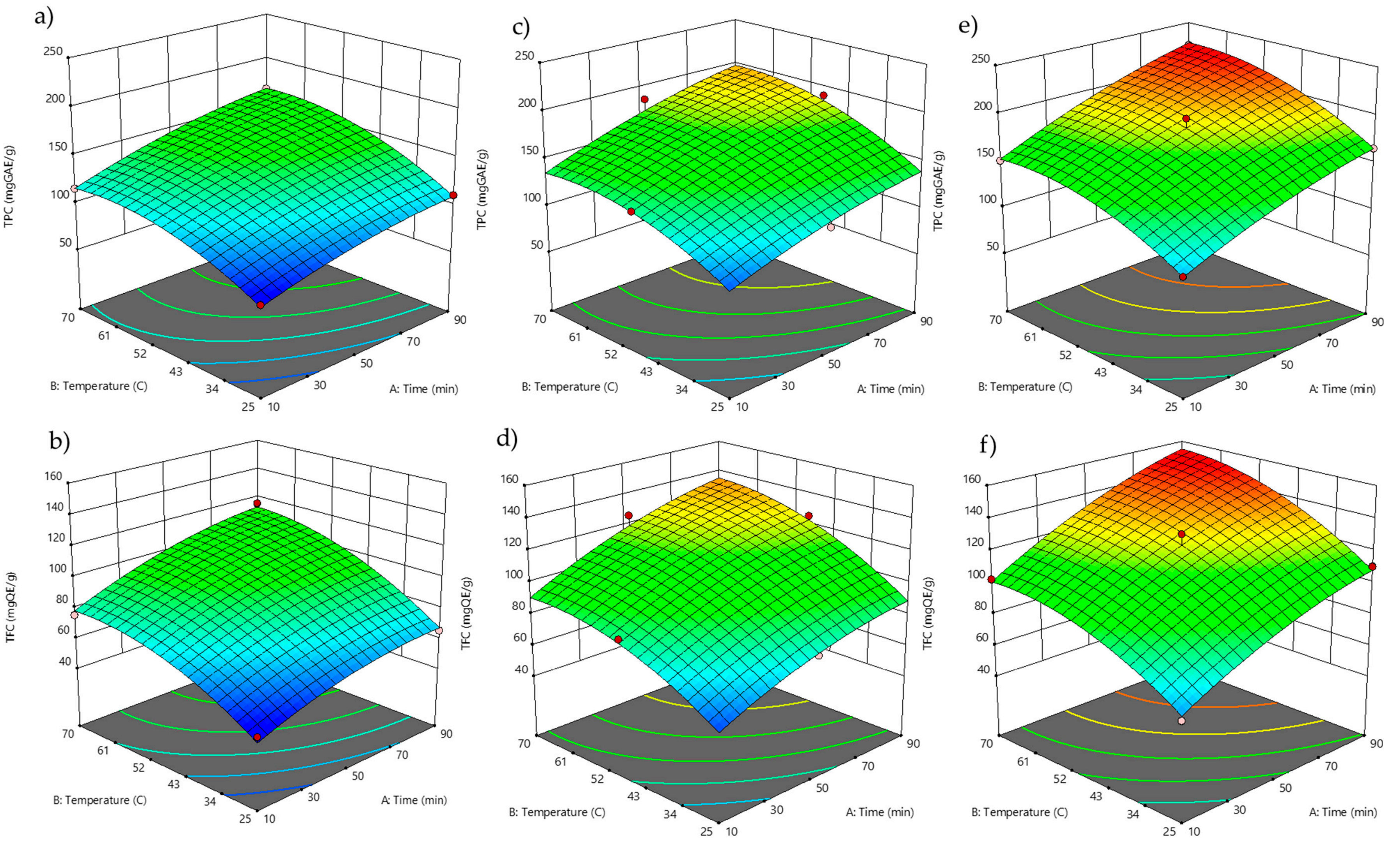

Figure S10. 3D response surface graphs for FRAP (mg AAE/g_{DW}) (a, c, e) and DPPH (%) (b, d, f) of rosehip ground leaves extracts after SLE as a function of temperature and time, at a S/L ratio of 0.05 g/ml, and at ethanol concentrations in water (v/v) of 0% (a, b), 40% (c, d), and 80% (e, f).

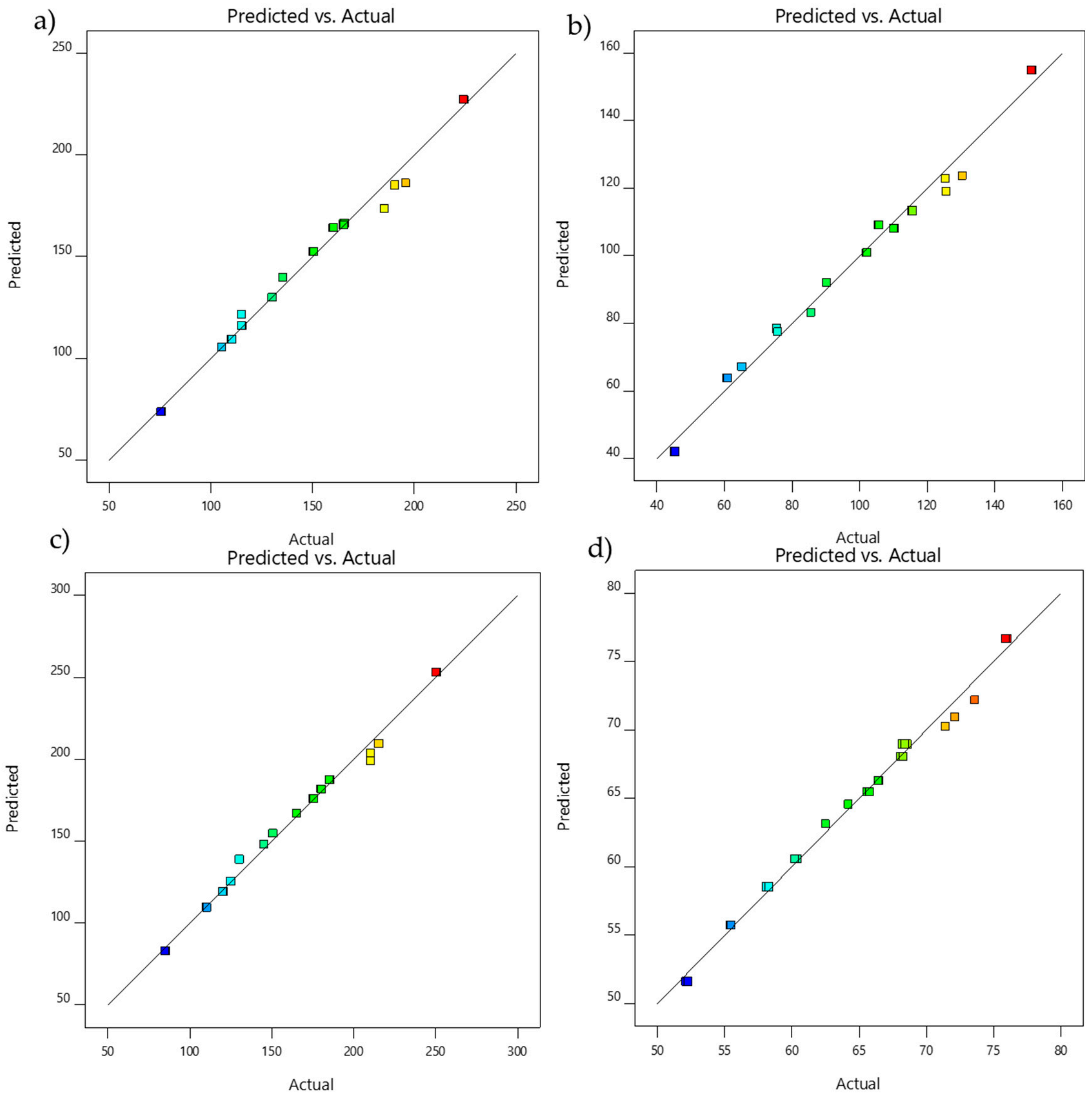

Figure S11. Predicted vs. actual values of TPC (a), TFC (b), FRAP (c), and DPPH (d) of rosehip ground leaves extracts obtained after SLE.

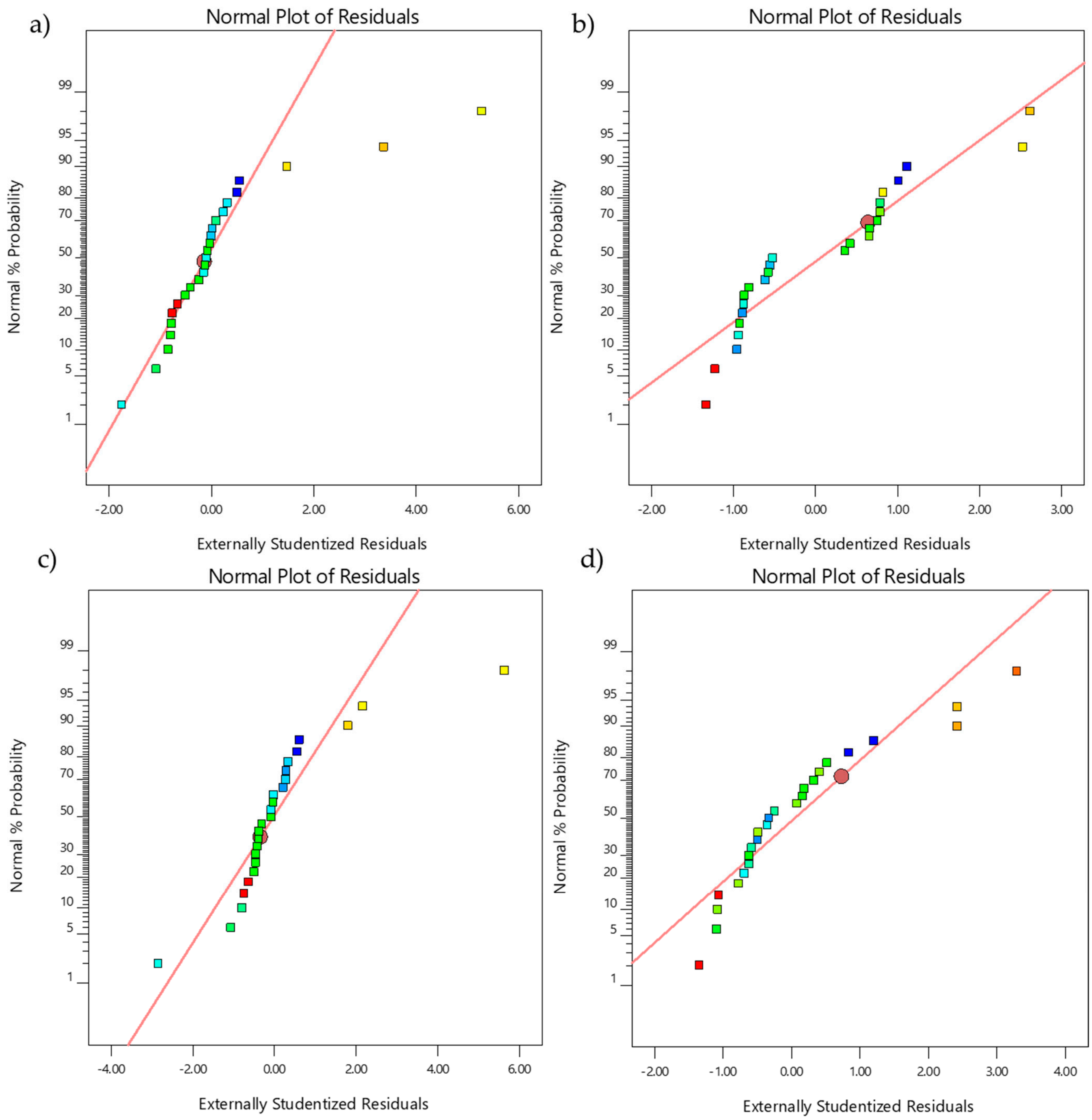

Figure S12. Normal plots of residuals for TPC (a), TFC (b), FRAP (c), and DPPH (d) of rosehip ground leaves extracts obtained after SLE.

Table S4. Second-order polynomial model coefficients for TPC, TFC, FRAP, and DPPH of rosehip ground leaves extracts obtained after UAE.

**Coefficients**								
	TPC (mgGAE/g_{DW})		TFC (mgQE/g_{DW})		FRAP (mgAAE/g_{DW})		DPPH	
α_0	-34.736	*	-18.420	*	-158.168	*	14.173	**
α_1 (time)	1.454	**	0.765	**	2.0625	**	0.302	**
α_2 (T)	3.507	***	2.223	***	7.3641	***	0.817	***
α_3 (EtOH)	1.787	**	1.281	**	1.9127	***	0.464	***
α_{12} (t x T)	-0.00167	ns	-0.0005	ns	-0.00133	ns	-0.000556	ns
α_{13} (t x EtOH)	0.0025	ns	0.00113	ns	0.00247	ns	0.0003	ns
α_{23} (T x EtOH)	0.00827	ns	0.00473	ns	0.0153	ns	0.00187	ns
α_{11} (t x t)	-0.00951	ns	-0.00510	ns	-0.0139	ns	-0.00186	*
α_{22} (T x T)	-0.0206	ns	-0.01486	ns	-0.0517	ns	-0.00532	ns
α_{33} (EtOH x EtOH)	-0.0175	*	-0.0135	**	-0.0206	ns	-0.00468	**
p value of the model	0.001	***	0.0004	***	0.002	**	0.0002	***
R^2	0.979		0.986		0.974		0.989	
Adjusted R^2	0.942		0.961		0.927		0.967	
Predicted R^2	0.823		0.879		0.788		0.916	
Lack of fit	F value: 0.150	ns	F value: 0.210	ns	F value: 0.080	ns	F value: 0.170	ns

ns not significant for $p > 0.05$

*Significant for $p \leq 0.05$; **significant for $p \leq 0.01$; ***significant for $p \leq 0.001$

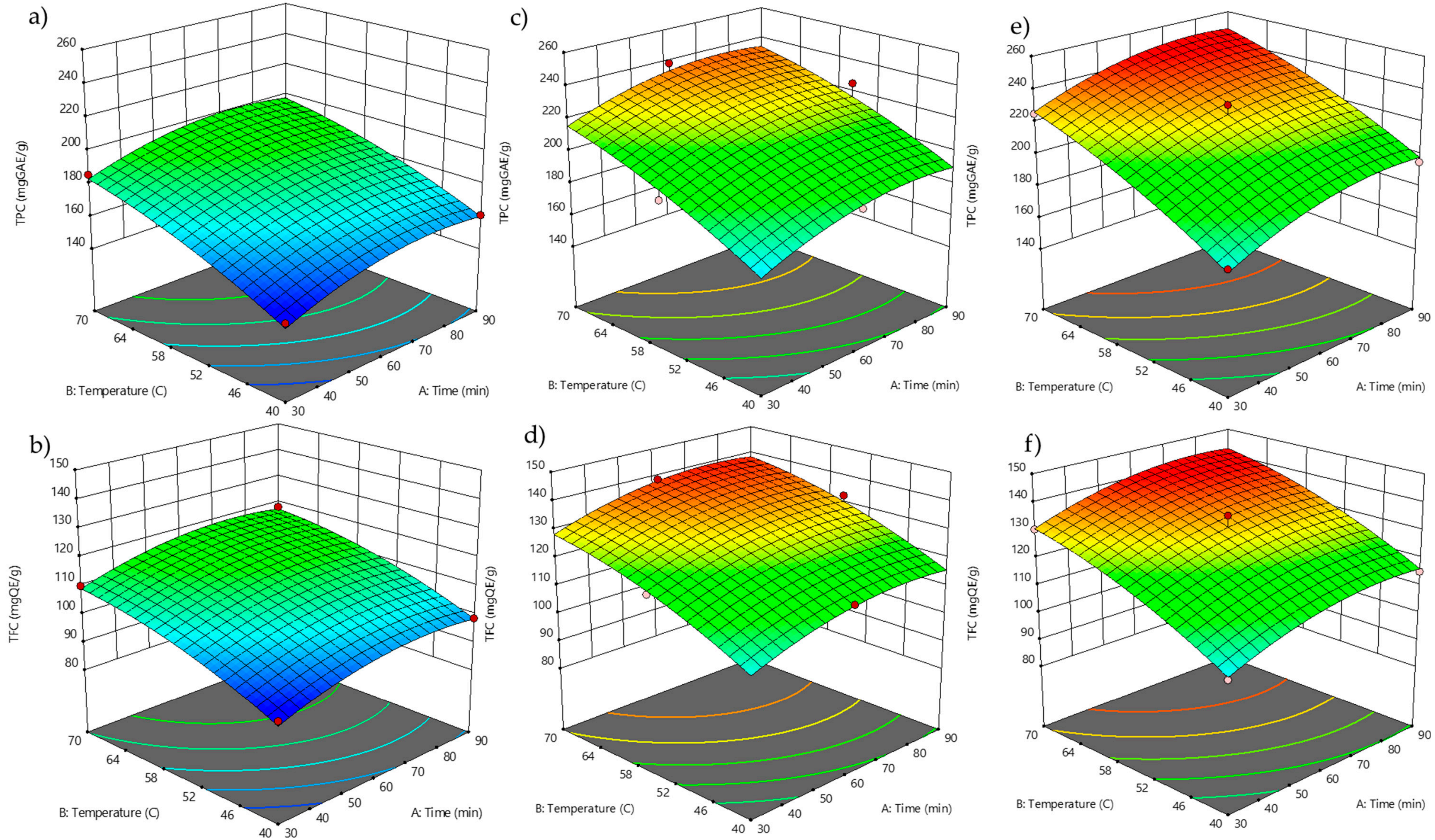

Figure S13. 3D response surface graphs for TPC (mg GAE/g$_{DW}$) (a, c, e) and TFC (mg QE/g$_{DW}$) (b, d, f) of rosehip ground leaves extracts after UAE as a function of temperature and time, at a S/L ratio of 0.05 g/ml, and at ethanol concentrations in water (v/v) of 20% (a, b), 45% (c, d), and 70% (e, f).

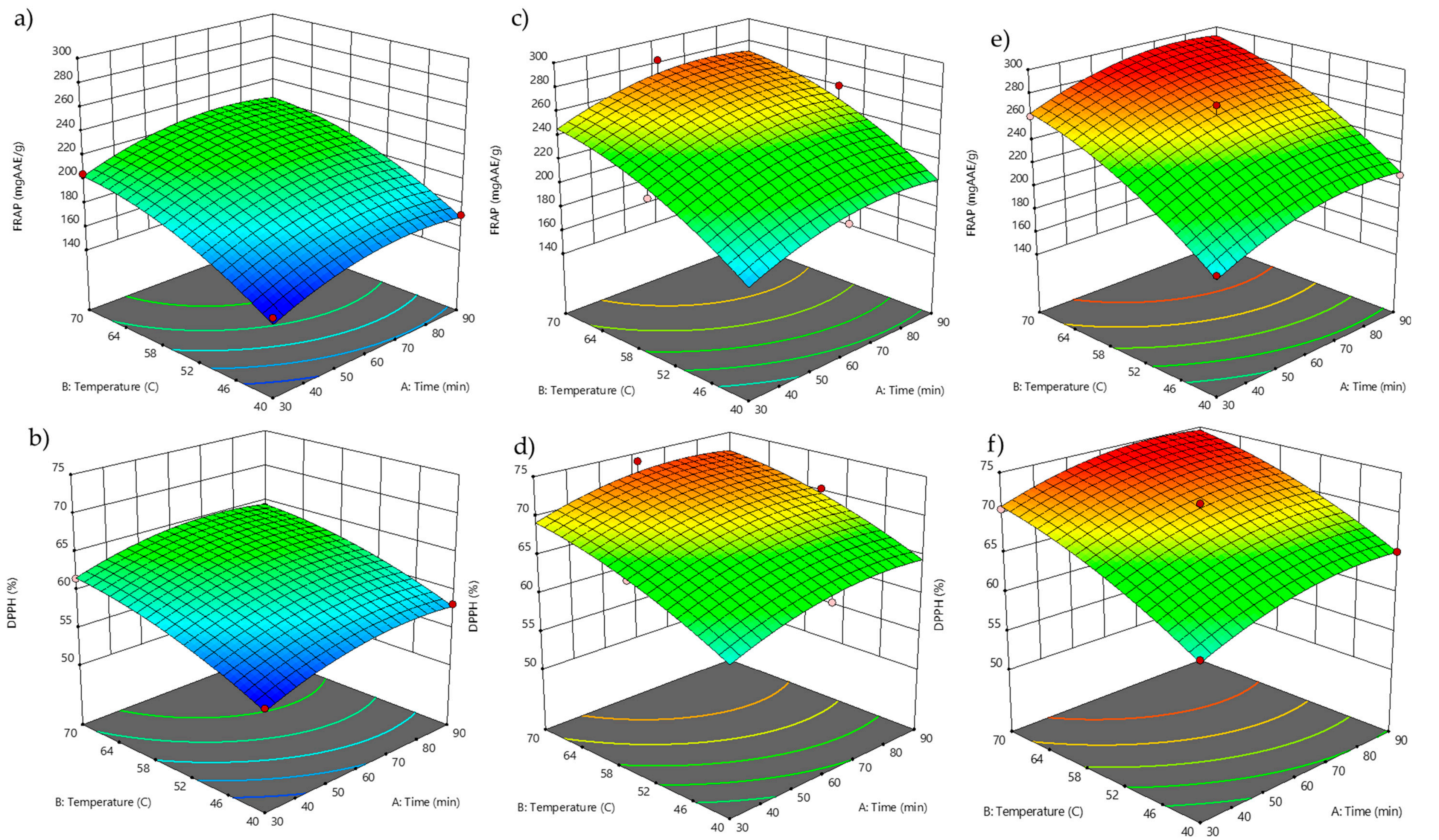

Figure S14. 3D response surface graphs for FRAP (mg AAE/g_{DW}) (a, c, e) and DPPH (%) (b, d, f) of rosehip ground leaves extracts after UAE as a function of temperature and time, at a S/L ratio of 0.05 g/ml, and at ethanol concentrations in water (v/v) of 20% (a, b), 45% (c, d), and 70% (e, f).

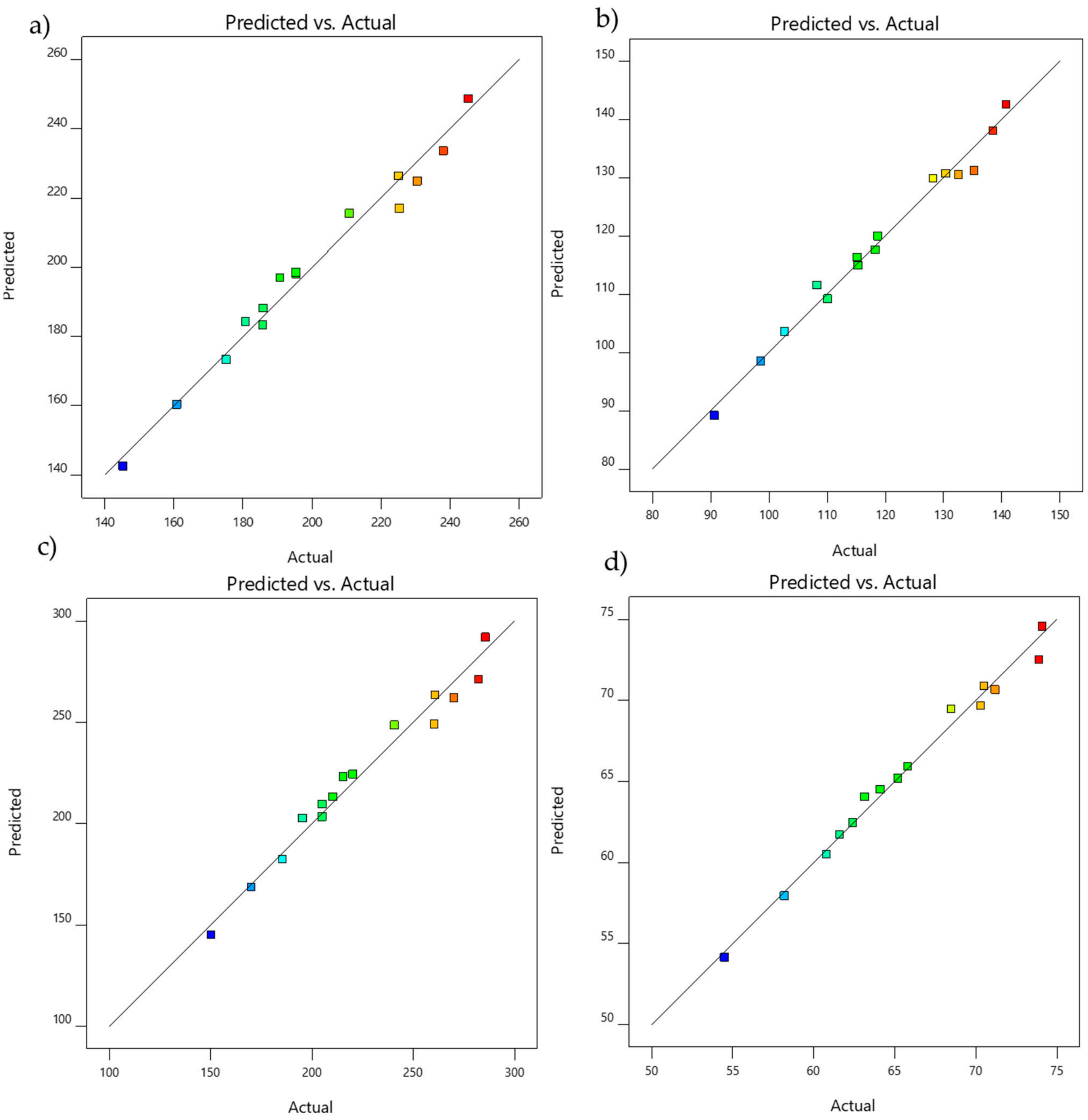

Figure S15. Predicted vs. actual values of TPC (a), TFC (b), FRAP (c), and DPPH (d) of rosehip ground leaves extracts obtained after UAE.

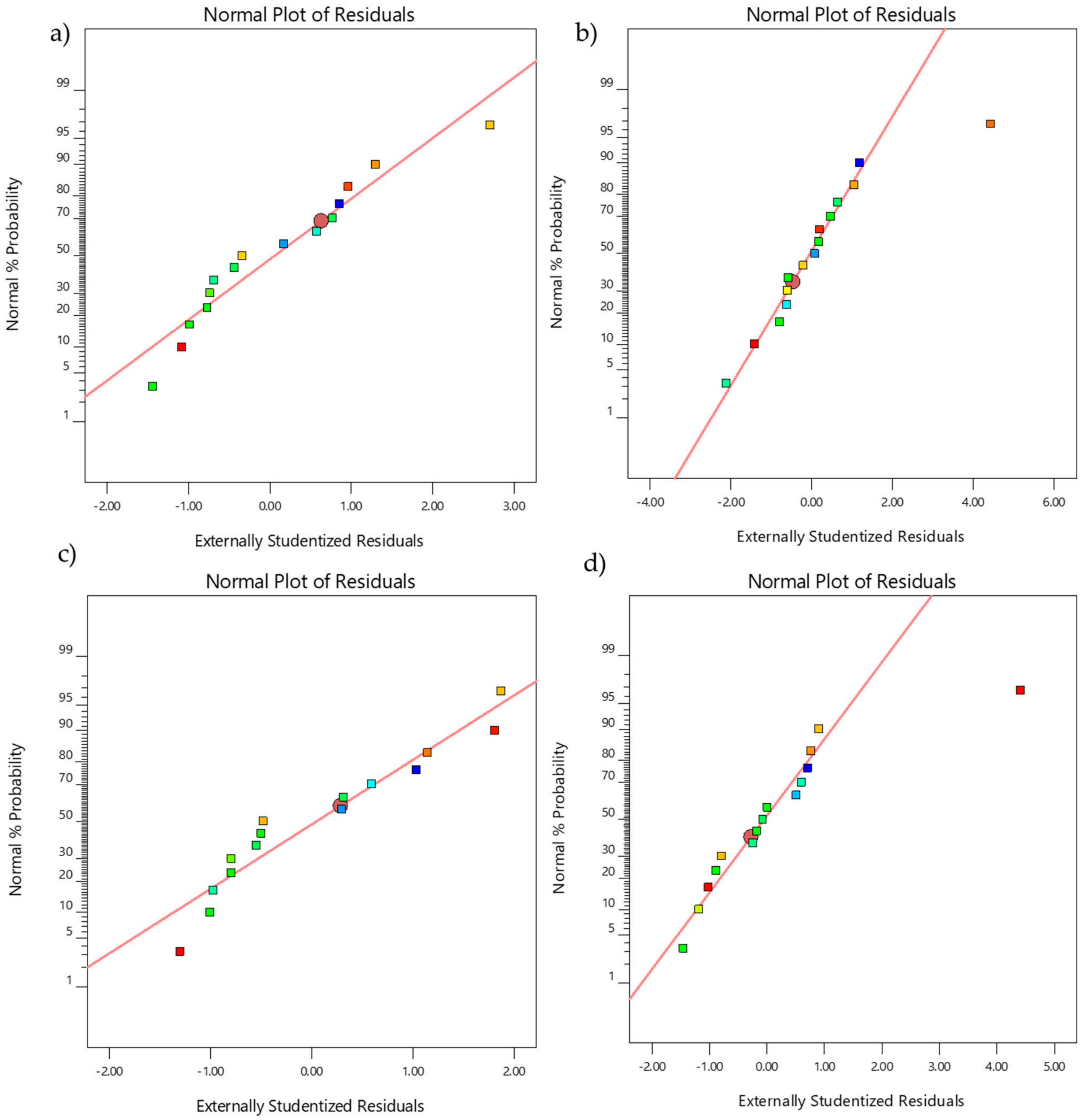

Figure S16. Normal plots of residuals for TPC (a), TFC (b), FRAP (c), and DPPH (d) of rosehip ground leaves extracts obtained after UAE.
